# Behavior and Potential Impacts of Metal-Based Engineered Nanoparticles in Aquatic Environments

**DOI:** 10.3390/nano7010021

**Published:** 2017-01-22

**Authors:** Cheng Peng, Wen Zhang, Haiping Gao, Yang Li, Xin Tong, Kungang Li, Xiaoshan Zhu, Yixiang Wang, Yongsheng Chen

**Affiliations:** 1 School of Civil and Environmental Engineering, Georgia Institute of Technology, Atlanta, GA 30332, USA; cpeng@dhu.edu.cn (C.P.); hgao49@gatech.edu (H.G.); xtong37@gatech.edu (X.T.); li.kungang@gmail.com (K.L.); 2 Department of Environmental Engineering, College of Environmental and Resource Sciences, Zhejiang University, Hangzhou 310058, China; 3 Department of Environmental Science, College of Environmental Science and Engineering, Donghua University, Shanghai 201620, China; 4 John A. Reif, Jr. Department of Civil and Environmental Engineering, New Jersey Institute of Technology, Newark, NJ 07102, USA; 5 State Key Laboratory of Water Environment Simulation, School of Environment, Beijing Normal University, Beijing 100875, China; liyang_bnu@bnu.edu.cn; 6 Shenzhen Public Platform for Screening and Application of Marine Microbial Resources, Graduate School at Shenzhen, Tsinghua University, Shenzhen 518055, China; zhu.xiaoshan@sz.tsinghua.edu.cn (X.Z.); 572997688@qq.com (Y.W.); 7 Joint Center for Global Change Studies (JCGCS), Beijing 100875, China

**Keywords:** aggregation, dissolution, ROS, toxicity, antibacterial, DNA interactions, DLVO theory, tropical levels, bioaccumulation, biomagnification

## Abstract

The specific properties of metal-based nanoparticles (NPs) have not only led to rapidly increasing applications in various industrial and commercial products, but also caused environmental concerns due to the inevitable release of NPs and their unpredictable biological/ecological impacts. This review discusses the environmental behavior of metal-based NPs with an in-depth analysis of the mechanisms and kinetics. The focus is on knowledge gaps in the interaction of NPs with aquatic organisms, which can influence the fate, transport and toxicity of NPs in the aquatic environment. Aggregation transforms NPs into micrometer-sized clusters in the aqueous environment, whereas dissolution also alters the size distribution and surface reactivity of metal-based NPs. A unique toxicity mechanism of metal-based NPs is related to the generation of reactive oxygen species (ROS) and the subsequent ROS-induced oxidative stress. Furthermore, aggregation, dissolution and ROS generation could influence each other and also be influenced by many factors, including the sizes, shapes and surface charge of NPs, as well as the pH, ionic strength, natural organic matter and experimental conditions. Bioaccumulation of NPs in single organism species, such as aquatic plants, zooplankton, fish and benthos, is summarized and compared. Moreover, the trophic transfer and/or biomagnification of metal-based NPs in an aquatic ecosystem are discussed. In addition, genetic effects could result from direct or indirect interactions between DNA and NPs. Finally, several challenges facing us are put forward in the review.

## 1. Introduction

The rapid development and expansion of nanotechnology industries have ultimately led to mass production of a wide variety of engineered nanoparticles (NPs) or nanomaterials (ENMs) that inevitably increase the possibility of release into the environment and exposure to ecosystems or even humans. These novel ENMs exhibit extraordinary performance in mechanical, electric, electronic, thermal and optical applications due to unique properties that traditional or bulk counterpart materials cannot begin to match. For instance, quantum dots (QDs), a type of semiconductor nanocrystal, possess remarkable optical and electronic properties that have been extensively used in experimental biomedical imaging, biolabeling and anti-counterfeiting applications to create special inks, dyes, paints, light displays and chemical sensing [1,2,3,4,5,6]. Metal oxide NPs, such as CeO_2_, ZnO and TiO_2_, have also been widely used in commercial products or industrial processes, such as sunscreens [7], antimicrobial agents [8,9], solar energy conversion [10,11] and photocatalysis for remediation of environmental pollutants [12,13]. With increasing and diverse applications, NPs will likely find their way into the environment through many pathways (Table 1) and further pose potential risks to the environment and human health. The sources of NPs released into the environment may comprise point sources (e.g., manufacturing facilities, landfills and wastewater treatment plants’ effluent) and nonpoint sources (e.g., storm-water runoff and wet deposition from the atmosphere) [14]. NPs may also be released at different nonpoint sources during their lifecycle; for example, NPs can be released due to abrasion or washing during use phases or through disposal and recycling at the end of their lifecycle. Accidental release during production or transport may also contribute to the release of NPs. On the other hand, intended applications also occur. One example is the release of NPs into the aqueous environments during in situ remediation of polluted natural waters using zero-valent iron NPs (ZVI NPs) [15].

**Figure 1:**
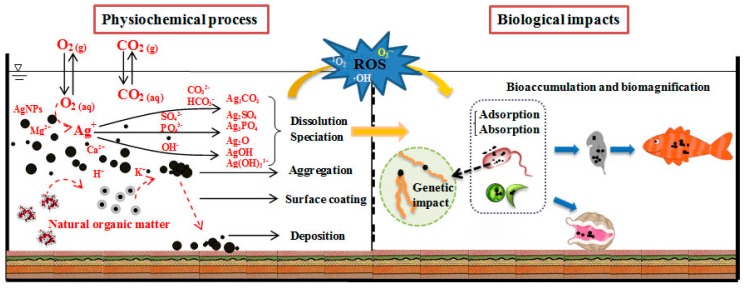
Potential physiochemical processes and biological impacts of metal-based NPs (e.g., Ag NPs) in natural waters (reprinted with major modification from [44] with permission, Copyright Elsevier, 2011).

Considering unintended or accidental spills or the release of ENMs into the environment, there is an increasing concern about ENMs’ effects on the environment, ecosystems and human health [27]. Many toxicological studies have indicated that many metallic NPs could be toxic to organisms, such as bacteria [28,29,30,31], algae [32,33], epithelium [34,35,36,37] and plants [38,39]. There are three pathways that induce the toxicity of NPs: (1) direct surface interactions or internalization of NPs; (2) release of toxic metal ions (e.g., Ag^+^ Cu^2+^ and Zn^2+^) from NPs; and (3) oxidative stress induced by ROS [21,40,41,42]. These adverse biological impacts were found to vary with material properties (e.g., size, shape, surface areas, chemical compositions, electronic properties, surface reactivity and functional groups or coatings) [43]. Furthermore, the complexities between material properties and toxicity are highly dependent on the environmental behavior of NPs. As illustrated by Figure 1, typical environmental behavior of NPs includes dispersion, aggregation, redox reactions, ion release or dissolution, speciation, complexation with natural organic matter (NOM) leading to surface coating, sedimentation and deposition onto solid phases, such as sediment or soil. These physicochemical processes, which could take place concurrently and influence each other, are sensitive to the properties of NPs. Meanwhile, the properties (e.g., size distribution, surface charge or hydrophobicity) of NPs are likely to be changed by the interactions of NPs with the environment. Moreover, both metal-based NPs and their released metal ions can adsorb into aquatic organisms and further be bioaccumulated and biomagnified in aquatic ecosystem. Thus, it is necessary to complement valuable mechanistic studies with systematic short-term and long-term animal experiments for providing predictable derived no-effect levels in the risk assessment of ENMs. The current situation is especially challenging, as testing capabilities, reliability and resources do not allow for an adequate assessment of ENM safety and risks due to knowledge gaps in the study of nanomaterial properties, such as uncertainties in their reactivity, transformation and behavior in aquatic environments.

This review summarizes research in the past few years that has investigated the aqueous behavior of metal-based NPs with in-depth analyses of the mechanisms, kinetics and subsequent environmental and ecological impacts. Specifically, we analyzed the potential portals for NPs to enter or exit the environment and discussed their estimated concentrations based on previous experimental and modeling studies. Kinetics and governing parameters (e.g., pH, salinity and NOM) for aggregation and dissolution of selected metal NPs were compressively evaluated with analytical methods and mathematical modeling. The mechanisms of ROS generation of different metal-based NPs and the quantitative relationships between the generated ROS concentrations by NPs were demonstrated to provide insight into cellular damage and oxidative stress on microorganisms. Finally, the impacts on aquatic organisms at multiple trophic levels, bacteria and single biomolecules (i.e., DNA) were extensively surveyed. The goals of this review were also to deliver the current level of knowledge about the safety of NPs or ENMs to identify knowledge gaps and to help prioritize research on the safety of ENMs.

## 2. Environmental Behavior of Metal-Based NPs in the Aqueous Environment

The high surface-to-volume ratio and resulting reactive properties make NPs highly dynamic in the environment. The environmental transformations may tremendously alter the physiochemical properties, fate, transport, reactivity, bioavailability and toxicity of NPs. A comprehensive understanding of the transformations of NPs in the environment can provide important insights into the potential environmental and human health implications of NPs.

### 2.1. Aggregation of Metal-Based NPs in the Environment

Of all of the transformations, aggregation of NPs in an aqueous environment has been one important aspect, as aggregation transforms NPs into several micron clusters in an aqueous environment, which not only alters the size distribution of NPs, but also alters NP transport characteristics and biological interactions. According to collision efficiency (also known as attachment efficiency, α), aggregation can be divided into two types of processes relating to NPs in the environment: (1) diffusion-limited cluster aggregation (DLCA); and (2) reaction-limited cluster aggregation (RLCA). When α is close to one, aggregation occurs in the DLCA regime, whereas RLCA becomes dominant when α < 1. Kinetics and aggregate structure in these two regimes are fundamentally different. In the RLCA regime, an increase in the electrolyte concentration screens surface charge and reduces the energy barrier to aggregation, which leads to faster aggregation. At electrolyte concentrations above the critical coagulation concentrations (CCC), the repulsive energy barrier will be eliminated, and rapid aggregation of NPs occurs in the DLCA regime. Recent studies apply the Derjaguin–Landau–Verwey–Overbeek (DLVO) theory to predict the aggregation behavior of NPs under various aqueous environmental conditions. Classic DLVO is based on the interaction energy balance that consists of attractive van der Waals (vdW) and repulsive electrostatic forces from the overlap of the electrical double layers (EDL) of interactive surfaces [45]. On the basis of the extended DLVO (EDLVO) theory and the von Smoluchowski’s population balance equation, different diffusion-limited aggregation (DLA) and reaction-limited aggregation (RLA) models were established to predict the aggregation kinetics of CeO_2_ NPs in solutions [46]. The predictions derived from the established models were in close agreement with the experimental observations. The effects of electrolyte valence and ionic strength on NP stability with respect to aggregation can be well interpreted by classic and extended DLVO theory [44]. The model is useful for pre-evaluation of aggregation tendency of NPs in the presence of NOM.

Nanoparticle aggregation strongly depends on the properties of primary NPs (e.g., particle size and shape, surface coatings, chemical composition), solution chemistries (e.g., pH, ionic strength, electrolyte patterns and NOM), and various environment conditions (e.g., temperature and dissolved oxygen) [47].

#### 2.1.1. Effects of Size and Shape on the Nanoparticle Aggregation

According to the DLVO theory, the interaction energy barrier decreases with decreasing particle size. The decrease in particle size results in the presence of a higher ratio of atoms on the particle surface, which alters the electronic structure, surface charge and surface reactivity [48]. Smaller particles with high surface energy can aggregate more readily than larger ones since the aggregation can reduce the free energy in the NP system.

Moreover, NPs may show many kinds of irregular shapes (e.g., nanowire, nanotube and nanorods), which are different from the spherical shapes of particles in DLVO modeling. Both vdW and EDL forces would be influenced by the change of shape [49]. Theoretically, EDL forces relate to interacting orientation for irregular particles, which results in different atomic arrangements of these particles on the surface. Some nonconventional theories, including surface element integration [50,51], may be used to explain the interfacial forces that cause the irregular shapes. Finally, the energies of vdW and EDL are different, which also affects the aggregation of NPs.

Crystal lattice defects as charge carriers will definitely alter the surface charge of NPs [52,53]. Even with the same composition, different crystal structures may result in different NP surface charges. For instance, TiO_2_ NPs with three phases of crystallinity, including rutile, anatase and brookite, have different surface charge, which can also affect aggregation and deposition rates [54]. This is because various crystallographic planes with different atomic densities can interface with the aqueous phase to produce different extents of EDL and surface energy. Shifts could be found in exposed crystal face compositions with rutile rod size changes [55]. Since different rutile crystal faces possess different surface energies [56], changes in exposed crystal face composition might lead to different degrees of surface energy and consequently affect colloidal stability.

#### 2.1.2. Effect of Surface Coating and Surface Hydrophobicity on the Nanoparticle Aggregation

Surface coated-NPs are typically stabilized via strengthening of electrostatic, steric or electrosteric repulsion between NPs; these forces consequently alleviate aggregation or provide other specific surface functionality, which has a stabilizing effect [57,58,59,60,61,62]. There are three kinds of typical surface coatings: surfactants, polymers and polyelectrolytes [63,64,65,66]. Adsorbed or covalently binding surfactants affect aggregation stability by increasing surface charge and electrostatic repulsion or by reducing interfacial energy between particles and solvent [67]. For example, sodium dodecyl sulfate (SDS), as a representative of the anionic surfactant group, is widely used to stabilize NPs against aggregation via affecting electrostatic repulsion [68]. Polymers like polyvinylpyrrolidone (PVP) display a stabilizing effect based on steric repulsion. For instance, the CCC value of PVP-Ag NPs is four times higher than that of bare Ag NPs [57]. Even under various chemical composition of the water matrix, the diffusion behavior of PVP-Ag NPs still remained unchanged [69]. The interaction between the steric repulsion and universal Coulomb attraction is caused by the surface coating layers, which may profoundly affect the aggregation kinetics. However, a recent study showed that sodium citrate had a more effective stability for spherical TiO_2_ NPs compared with PVP, SDS and polyethylene glycol (PEG) due to the lower CCC value [70]. Moreover, poly(diallyldimethylammonium chloride) (PDDA), one of the polyelectrolytes, is a cationic polymer that can protect NPs from oxidation and agglomeration due to its reducing and stabilizing function [71,72]. In addition, gum arabic-coated Ag NPs (GA-Ag NPs) have stronger stability than that of alginate-coated Ag NPs (ALG-Ag NPs) and natural polysaccharide-coated Ag NPs [73].

The properties of surfactants, such as chain length, molecular weight, types of head groups and the affinity of coating molecules for particle surfaces, can significantly affect the adsorbed surfactant mass and layer conformation, which in turn can affect the ability of a surfactant to enhance NPs’ aqueous dispersion stability [74,75]. Dederichs et al. found that the chain length of a surfactant is linearly related to the logarithm of the dispersion concentration, which defines the lowest concentration of a surfactant necessary to disperse hydrophobic particles [76].

Surface hydrophobicity or wettability can affect aggregation via changing the Hamaker constant (*A*_H_). *A*_H_ indicates the strength of the long-range mutual attraction between two interacting materials, which governs vdW attraction [77]. Particles with a high *A*_H_ have a greater aggregation tendency compared to particles with a low *A*_H_ under the same surface and solution chemistry.

#### 2.1.3. Effect of Solution Chemistry on the Nanoparticle Aggregation

Studies to date have addressed different solution pH and ionic strength effects on the aggregation of various NPs in aqueous solutions [36,78,79]. pH and ionic strength (quantification of dissolved ionic species) influence NP stability in aqueous environments, primarily because these two factors determine their surface charge and charge density to a large extent. Most metal NPs contain surface functional groups, such as oxide and hydroxide groups, which can be associated with H^+^ or OH^−^ under aqueous conditions. Since most surfaces are negatively charged at circumneutral pH in the environment, this surface charge reversal would significantly affect NP aggregation due to the decreasing electrostatic repulsion and the vdW attraction prevailing around the pH of a zero surface charge. Aggregation of ZnO NPs indicates pH dependence, which is in agreement with the reported point of zero charge (pH_zpc_ 9.2). Guzman et al. noted in their study of TiO_2_ NPs that as the solution pH approached pH_zpc_, there was an observed increase in the hydraulic diameter of NPs within the aggregates [80].

With respect to the ionic solute effect, the elevated ionic concentration leads to a decrease in the extent of EDL repulsion, which means the electrostatic energy barrier is reduced; thus, aggregation can be promoted. Electrostatic destabilization is strongly affected by the valency (*z*) of ions [53]. The valence increase leads to the inverse of the Debye length increase, which results in lower repulsive electrostatic energy, which will likely enhance aggregation. When the ionic concentration approaches the CCC, the repulsion energy barrier will be completely eliminated, and rapid aggregation will occur.

Alginate-coated hematite NPs underwent aggregation via electrostatic destabilization in the solution with NaCl and MgCl_2_, which corresponded to the DLVO theory, whereas the aggregation rate was much higher in the presence of MgCl_2_. French et al. found that aggregation of TiO_2_ NP occurred faster in solution containing divalent cations (e.g., Ca^2+^) than in those containing monovalent cations, such as Na^+^, at the same pH and ionic strength [81]. The CCC can be estimated to be inversely proportional to the sixth power of metal ions’ valency, which is known as the Schulze–Hardy rule. Compared with monovalent ions, such as Na^+^, the CCC for a specific solution with the presence of divalent ions (e.g., Ca^2+^) should be much lower. The lower CCC value demonstrates destabilization and higher aggregation potential. For example, TiO_2_ NPs were significantly aggregated in the range of 1–500 mM NaCl and 0.05–40 mM CaCl_2_ solutions. Especially in the divalent electrolyte solution, TiO_2_ NPs had a considerably lower CCC (1.3 mM) [82]. In contrast, bovine serum albumin (BSA)-NP conjugates became more stable with increasing ironic strength due to the enhanced steric force and the shield of the attractive patch-charge force [83].

#### 2.1.4. Effect of NOM on the Nanoparticle Aggregation

NOM, which is mainly comprised of humic and fulvic substances, is ubiquitous in natural aqueous environments. NOM is expected to attach to the surface of NPs, changing the physiochemical properties of NPs and the interfacial forces or energies between interacting NPs, thereby altering the aggregation behavior [84]. The physical structures and molecular weights of NOM can change with solution chemistry, such as pH, ionic strength and electrolyte type [85]. As discussed previously, there is an increase in the aggregation of ZnO NPs with the increasing ionic strength. However, this trend could go in the opposite direction with the addition of humic acid (HA) (up to 3 mg·L^−1^). NPs coated with NOM had slow aggregation even at high ionic strengths [86]. The effect of HA on NP aggregation is related to ionic strength and electrolyte type. It was found that HA exhibited a stabilizing effect on Au NPs at low ionic strength and in the presence of monovalent cations; however, elevated concentrations of divalent ions lead to enhanced aggregation [87]. For instance, at low concentrations (0.004 M) of CaCl_2_, HA will inhibit aggregation of CeO_2_ NPs; however, HA will enhance aggregation at high concentrations (0.08 M) of CaCl_2_. The sedimentation rate of ZnO NPs was found to be faster at the lowest dissolved HA concentration (1.7 mg·L^−1^) than the sedimentation rate in the absence of HA [78]. In addition, the influence of HA on the NP agglomeration strongly depends on the reaction time [88].

It was determined that HA can affect the stability of NPs via steric hindrance, charge neutralization and bridging effects [89]. For instance, TiO_2_ NP aggregation was enhanced with the addition of HA only when the ionic strength was lower than CCC (75 mM) and the pH was less than the pH_zpc_ (pH 6) because HA reduced the zeta potential of TiO_2_ NPs through charge neutralization. When the ionic strength is higher than CCC, HA can promote aggregation of TiO_2_ NPs via the bridging effect. When the ionic strength is lower than CCC, aggregation can be inhibited due to steric hindrance by adding HA. Generally, the zeta potential can be used to predict the interaction forces between interacting particles. Zeta potential measurements have indicated that the surface charge of NPs is sensitive to ionic strength in the absence of HA. Both NaCl and CaCl_2_ addition results in smaller zeta potential values of NPs, and divalent cations are more effective, leading to faster aggregation. In contrast, the increase of ionic strength has much less influence on the surface charge of NPs in the presence of HA, where the combined electrostatic and steric repulsion may make NPs stable. In the presence of Ca^2+^, the divalent cations neutralized the negative surface charge of NPs imparted by NOM and thus induced aggregation. Different NP capping agents (e.g., anionic agent citrate acid and PVP) are thought to play a role in the colloidal stability of NPs. However, in the presence of NOM, the adsorption of NOM is the predominating factor, not the capping agents.

Given the complex compositions of NOM, it is still important to further understand how specific components in NOM affect the aggregation behavior of NPs [85,90,91]. The aggregation rate of ZnS NPs was observed to decrease with the increase of the aromatic content and the average molecular weight of NOM. When separating Suwannee River NOM fractions using ultrafiltration membranes into filtrate NOM (NOM_f_) and retentate NOM (NOM_r_), 10 mg·L^−1^ of NOM_f_ inhibited the aggregation of Au NPs compared with that without NOM [85]. In contrast, 10 mg·L^−1^ of NOM_r_ stabilized Au NPs significantly [85]. Four other NOM isolates (i.e., small and large molecular weight Suwannee River HA (SRHA), Suwannee River fulvic acid (SRFA) and Pony Lake FA) were investigated, and all four types stabilized the citrate-coated Au NPs (CIT-Au NPs) in terms of aggregation, whereas different NOM isolates showed various effects [91]. A recent study reported that at high ionic strength (100 mM NaCl), the molecular components of NOM whose weight fraction was higher than 100 kg·mol^−1^ with more aromaticity provided better stability against NP aggregation than molecular components at lower ionic strength [84]. This effect can be explained by steric repulsion mechanisms. Moreover, compared with the separated components, unfractionated NOM provided better stability, which suggests that there is a synergistic effect between the high and low molecular weight fractions for NP stabilization [84]. The types and compositions of NOM likely vary in electrostatic and steric repulsion and thus influence the stability of NPs in aqueous environments.

As a part of NOM, non-humic substances, mainly including biological macromolecules, can also alter the NP stability by changing the NP surface charge [83]. The presence of extracellular polymeric substance (EPS) dramatically decreased the aggregation rate and increased the CCC values of TiO_2_ in the NaCl solution via steric repulsion, but enhanced aggregate growth at a high CaCl_2_ concentration via intermolecular bridging to link the TiO_2_ NPs and aggregates [82]. The addition of cytochrome c increased the CCC value of hematite NPs in the NaCl solution due to the electrosteric repulsion, whereas BSA accelerated the NP aggregation even at low ironic strength via the strong attractive patch-charge interaction [83]. In addition, the presence of cystine intensified the aggregation of citrate-Ag NPs (CIT-Ag NPs), but with a shift in the CCC to lower cystine concentrations due to the charge and sterical stabilization [92].

#### 2.1.5. Effect of Solution Temperature and Dissolved Oxygen on the Nanoparticle Aggregation 

Temperature is an important environmental factor that can alter the NP aggregation kinetics. However, few studies have investigated temperature as it relates to NP aggregation [93,94]. Grasso et al. reported that temperature affects aggregation kinetics through influencing random Brownian motion of particles [85], and Nel et al. discussed temperature and NP collision frequency [95,96]. High temperature enhances the collision frequency between particles via increasing the random kinetic energy of NPs [97]. Thus, the aggregation rates increase substantially with the increasing temperature [39]. However, some research indicated that the zeta potential became less positive when the temperature increased, because increasing temperature promotes proton desorption from the particle surface and further reduces the electrostatic repulsion force or energy barrier between particles [98,99]. Dissolved oxygen (DO) in an aquatic environment results in the oxidation of metallic NPs, such as Ag NPs and QDs; furthermore, rapid surface oxidation has demonstrated its influence on the aggregation process. However, research related to the DO effect on the aggregation of NPs is limited. Ag NPs not only aggregated, but also released Ag^+^ as a result of oxidation [46]. In the presence of DO, the hydrodynamic diameters became a random distribution and fluctuated periodically, but without DO, the hydrodynamic diameters of Ag NPs increased stably and linearly. The Ag NP aggregation rate with DO of 7.8 mg·L^−1^ was 3–8 times faster than that without DO depending on the different sizes of Ag NPs. DO presented substantial effects on the Ag NP aggregation rate due to the release of Ag^+^ [100]. Ag^+^ released into the solution not only increased the local ionic strength that compressed the EDL and reduced electrostatic repulsion, but also changed the surface from zero valent Ag (Ag^0^) to Ag_2_O and, consequently, affected aggregation.

### 2.2. Dissolution of Metal-Based NPs in the Aquatic Environment

Dissolution can play a critical role in the fate, behavior and toxicity of metal-based NPs in an aquatic environment. The toxicity of NPs to organisms partly results from the metal ions released from metal-based NPs, such as ZnO NPs and Ag NPs [101,102]. For example, CdSe/ZnS QDs can release toxic ions, including Cd^2+^, SeO_4_^2−^ and Zn^2+^, due to the diffusion of the core-shell structure [28]. Ag NPs that released Ag^+^ can even completely dissolve in aqueous environments in the long term [103]. ZnO NPs can rapidly dissolve in water to form hydrated Zn^2+^ cations [104,105]. The dissolution is also a common nature for CuO NPs, Fe_2_O_3_ NPs and SiO_2_ NPs [106,107]. TiO_2_ NPs are regarded as relatively stable and almost insoluble in aquatic systems. However, dissolved titanium in aqueous NaCl solutions have been detected [108]. Moreover, the dissolution of some general insoluble rare oxide particles at the nano-level can also be observed. Therefore, not only the properties of metal-based NPs, but also the environmental factors have impacts on the dissolution kinetics of metal-based NPs in an aquatic environment.

Dissolution depends on the solubility of materials in certain solvents and the concentration gradient from the solute surface and bulk solution [109]. The thermodynamics of material dissolution is described by a modified Kelvin equation (Ostwald–Freundlich relation) [62], which states that the solubility of NPs may increase exponentially with the particle size. On the other hand, the dissolution rate of a solute will increase dramatically in a short time before the rate decreases and the concentration in the bulk solution finally reaches equilibrium. Dissolution kinetics was previously evaluated by experimental and modeling approaches with the Arrhenius equation to interpret the effect of oxygen, protons and temperature on the release rate of Ag NPs [103]:

(1) $$\gamma_{{\mathrm{Ag}}^{+}}=\frac{3}{4}{\left(\frac{8\pi k_{B}T}{m_{B}}\right)}^{1/2}\;\rho^{-1}\;\exp(\frac{-E_{a}}{k_{B}T})[\mathrm{Ag}]r^{-1}{\left[O_{2}\right]}^{0.5}{\left[H^{+}\right]}^{2}$$

where γ is the release rate of Ag^+^ (mol·L^−1^·h^−1^); reactant *B* is either oxygen or protons; *k*_B_ is the Boltzmann constant (1.38 × 10^−23^ J·K^−1^); *m*_B_ is the molecular weight of reactant *B* (g·mol^−1^); ρ is the density of Ag NPs; *E*_a_ is the activation energy (J); *T* is temperature (298 K); [Ag] is the mass-based concentration of Ag NPs (μg·L^−1^); [O_2_] and [H^+^] are the molar concentrations (mol·L^−1^) of dissolved oxygen and protons, respectively; and *r* is the Ag NP radius (nm). The model shows that the release rate of Ag^+^ is dependent on *T*, [Ag], [O_2_] or [H^+^] and is inversely proportional to particle size (*r*).

#### 2.2.1. Effect of Primary Particle Size and Shape on the Nanoparticle Dissolution

A small particle size can always facilitate the rate of particulate dissolution. It has been reported that small Ag NPs (20 nm) took longer than large NPs (80 nm) in Hoagland medium to reach reaction equilibrium [103], which agrees with other studies that Ag NP dissolution shows strong size dependence [75,110,111]. Similarly, with the reduction of CuO NP size, there is a significant increase in dissolution rate and equilibrium concentrations [112,113]. The same size effect was also reported for the dissolution of QDs [114,115]. In a long dissolution time, the amounts dissolved from TiO_2_ NPs (28.3 nm) were smaller than those sized 4.7 nm [108]. Because the decreased size can increase the specific surface areas and the enthalpies of formation, the solubility of NPs is higher than that in the bulk phase [116,117]. Nonetheless, the size effect on the dissolution of ZnO NPs is not so obvious even between NPs and bulk or large-sized particles due to the high solubility of ZnO, which can reach up to 80% dissolution [78,101,113,118].

The shape of NPs was shown to influence both the equilibrium concentrations and rates of their dissolution. For instance, the Cu released from spherical, rod and spindle CuO NPs went up to 2.5%, 1.1% and 0.8% (wt % of NPs) within 72 h, respectively [119]. An apparent equilibrium Cu concentration was observed within 24 h for spherical CuO NPs, while for rod CuO NPs, it was reached after 60 h [112]. The dissolved rate of spherical CuO NPs was faster than that of rod and spindle CuO NPs [119]. Similarly, both wire and rod Pt nanomaterials showed higher resistance to dissolution than spherical NPs [120]. The dissolution rates and equilibrium concentration discussed thus far can be traced to shape-related differences in the anisotropic structure, specific surface area and the suspension stability induced by the differences in NP dissolution rates and the diffusion of metal ions in solution [112,120,121,122]. Moreover, due to the lower coordination numbers, the {1 1 1} and the {1 1 0} faces dissolved more rapidly than the {1 0 0} faces on PbS nanocrystals [123]. Meanwhile, the surface dissolution of NPs whose faces were adjacent (1–2 nm or less) to other NPs was significantly inhibited, which was attributed to the altered properties of aqueous solution and ion transport in confined spaces [123,124]. Using high-speed atomic force microscopy (AFM), Hoshi et al. found that the cubic Pt NPs were dissolved from the edge, while the edge of cuboctahedral Pt NPs and the top of tetrahedral Pt NPs were dissolved, forming terrace-like structures [125].

#### 2.2.2. Effect of Surface Coating on the Nanoparticle Dissolution

Dissolution of NPs clearly is affected by surface coating of NPs. Quite often, the release rate of most metal-based NPs decreases significantly in the presence of surface coating. Under the same environment conditions, bare-Ag NPs dissolved most easily compared to the coated Ag NPs [30]. The coated NPs exhibited less dissolution because the surface coating acts as a physical barrier or shield, preventing electrons or photons from transferring to the NP surface [75]. Even in the high NaCl solution (1 M), both the alginate- and gum arabic-coated Ag NPs had a low dissolution, which less than 10% of total Ag [73]. However, compared to bare ZnO NPs, organic coating delayed the dissolution equilibrium, but led to an increased concentration of Zn ions at equilibrium [126]. In addition, the composition of coating agents can have different effects on the dissolution of NPs. Li et al. found that electrostatically-stabilized CIT-Ag NPs dissolved faster than sterically-stabilized PVP-Ag NPs [30]. Yang et al. also reported that PVP-Ag NPs contained 1.6% dissolved silver, while CIT-Ag NPs contained 0.1% with the same size, probably due to the availability of citrate to reduce dissolved silver via chelation [102]. Tween 80 (polysorbate 80) inhibited dissolution of Ag NPs better than the SDS due to the thicker and more rigid Tween coating layer [75]. The dissolution rate of polydiallyldimethylammonium chloride (PDDA)-coated QDs was lower than that of poly(ethylene glycol) (PEG)-coated QDs due to the higher chain length and structural complexity of PDDA [28]. As for modified iron oxide NPs, only the citrate-iron oxide NPs released free iron ions in the 14-day test, and dextran-iron oxide NPs dissolved slowly in one-year-aged solutions, but the free iron could not be checked in the solutions of ascorbate-iron oxide NPs and PVP-iron oxide NPs [127].

#### 2.2.3. Effect of Solution pH, Electrolyte and Redox Conditions on the Nanoparticle Dissolution

As shown in Equation (1), the dissolution kinetics of metal NPs are affected by pH [103]. The solubility of Ag, QDs, ZnO, CeO_2_, Cu and CuO NPs is enhanced with the decreasing pH [75,113,128,129,130]. The addition of electrolytes varies the dissolution of NPs due to the potential chemical reactions and impacts on aggregation states. After the addition of electrolytes, Ag NPs dissolved immediately due to electrolyte-induced perturbations [131,132]. Moreover, the dissolution of Ag NPs strongly depends on the types and concentrations of electrolytes. When NaCl as a electrolyte was introduced in the aquatic system, the released Ag^+^ would combine with Cl^−^ to form AgCl precipitate as a coating layer, and then, the dissolution rate might be decreased [132]. The enhanced NPs’ release rate after replacing NaCl with NaNO_3_ can be attributed to both the perturbation in solution and the nitrate-promoted oxidative corrosion [132]. ZnO NPs released most Zn ions in seawater with a higher ionic strength as compared to fresh water [133]. However, the dissolution of Tween-coated Ag NPs was higher in NaCl than in NaNO_3_, which might be caused by the nucleophilicities difference between Cl^−^ and NO_3_^−^ ions [75]. Additionally, little change on the solubility of CuO NPs can be found before and after the addition of NaCl to deionized water [121].

Anaerobic or aerobic conditions are significantly different in the way they influence the dissolution of metal NPs [134]. One example is that the released contents of Cd and Se are much higher in air-saturated water than in the anaerobic condition [28]. In contrast, the release of Ag^+^ from the Ag NPs in deoxygenated water was terminated even at the lowest pH as opposed to the increased dissolution of the air-saturated condition. Clearly, dissolution of metal NPs is a cooperative oxidation process involving both dissolved oxygen and protons [75,135]:

(2) $${\mathrm{Me}}_{\left(s\right)}+\frac{n}{4}O_{2(\mathrm{aq})}+n{H^{+}}_{\left(\mathrm{aq}\right)}\Leftrightarrow {{\mathrm{Me}}^{n+}}_{\left(\mathrm{aq}\right)}+\frac{n}{2}H_{2}O$$

where Me and *n* are the metal element and the charge number of Me. 

#### 2.2.4. Effect of NOM on the Nanoparticle Dissolution

Our previous study revealed that at a low concentration of HA, the release process of Cd and Se from QDs was facilitated due to the sensitization effect of HA, whereas when the HA concentration was up to 50 mg·L^−1^, the release rate of Cd was reduced because of the complexation of HA and metal ions [28]. The presence of dissolved NOM such as SRFA promoted the dissolution of CuO NPs and Ag NPs in aquatic environments resulting from the enhanced stability via adsorption of NOM onto the Ag NP surface and the displacements of electrostatic surface coating [33,69]. However, in the presence of SRFA, the release rate of Zn^2+^ from ZnO NPs slowed down, and their dissolved concentration was slightly decreased [129]. Furthermore, SRFA and Pahokee peat fulvic acid (PLFA) reduced the dissolution of sulfidized Ag NPs [136]. It was speculated that SRFA may have dual roles on the dissolution of metal-based NPs [129]. On the one hand, SRFA was adsorbed on the NPs surface by electrostatic attraction and ligand exchange, so that the metal ions’ release was blocked [137]. On the other hand, the complexation of metal ions and SRFA caused more metal ions to be liberated into the bulk media [129]. Besides, for Ag NPs, humic/fulvic acids played the role of reductants to reduce the released Ag^+^ to Ag^0^ in a reversible reaction [138]. Furthermore, the presence of citric acid clearly promoted the release extent of ZnO NPs, which was caused by the interaction between complexing ligands and NPs, including the polarization and weakening of the metal-oxygen bonds of NPs [139]. Similarly, the amino acids and peptides in the culture medium accelerated the release rate of ZnO NPs [105]. For instance, ZnO NP dissolution was significantly accelerated, and its solubility was enhanced by cysteine [129]. Gondikas et al. also reported that the presence of cysteine increased the dissolution of coated Ag NPs. The sulfhydryl group of cysteine plays a vital role in the reactions [140]. Both the surface complexation and solution coordination induce the increased dissolution of NPs [129].

#### 2.2.5. Effect of Inorganic Species on the Nanoparticle Dissolution

Other inorganic ions or groups, such as phosphate (PO_4_^3−^) and sulfide (S^2−^), can still change the dissolution of metal-based NPs via some chemical reactions. For example, the release concentration of Zn^2+^ in solution decreased dramatically with a low concentration of added phosphate [141]. Similarly, the release rate of CeO_2_ NPs was inhibited in the presence of phosphate [130], which could complex with heavy metals ions [141]. In the presence of Na_2_S, the dissolution of Ag NPs was inhibited because the formation of sulfuric coatings can suppress the release of Ag^+^ from the surface of Ag NPs [122]. Yet, Ag NPs are rapidly dissolved with the addition of OCl^−^ [142].

#### 2.2.6. Effect of Temperature and Light Irradiation on the Nanoparticle Dissolution 

Temperature also affects the dissolution rate of NPs. The dissolution rate of QDs for both Cd and Se increased significantly when the temperature increased from 0 °C to37 °C, which was attributed to the accelerated mass transfer of dissolved oxygen and the reduced reaction activation energy by high temperature [28]. The temperature influence on the dissolution kinetics of NPs is shown in Equation (1) relating to the Arrhenius-based kinetics model [103]. 

Light exposure should be considered as another important environmental factor. Light irradiation can promote surface oxidation so that the physicochemical processes of metal-based NPs may be changed in aquatic environments [30]. Ultraviolet (UV) enhanced the release rate of Ag NPs and shortened the equilibration time [143], but no significant influence on the Ag release was observed after Ag NPs were exposed to sun light [61]. We found that the dissolved Ag^+^ concentration clearly increased with the irradiation exposure when compared to their response in a dark condition. Moreover, higher irradiation intensity obviously induced increasing release rates of QDs for both Cd and Se [28].

### 2.3. ROS Generation by Metal-Based NPs in the Aqueous Environment

One of the major toxicity mechanisms of metal-based NPs is related to the generation of ROS and subsequent ROS-induced cell damage or injury [29,43,105,144,145,146]. Many previous studies have demonstrated that three types of ROS, including singlet oxygen (^1^O_2_), hydroxyl radical (·OH) and superoxide radical (O_2_·^−^), may jointly contribute to and enhance the cytotoxicity of metal-based NPs in water [144,147,148,149]. Among these ROS, rather long-lived ^1^O_2_ (3.8 μs in water) is the most detrimental to cells because it reacts broadly with amine acids, such as methionine, vitamins, such as beta-carotene, unsaturated fatty acids, proteins and steroids [150,151], causing biomembrane oxidation and degradation [152]. Similarly, although short-lived (10 μs in water), ·OH is highly reactive and can nonspecifically oxidize virtually all types of macromolecules, including carbohydrates, nucleic acids, lipids and DNA [150,153]. Although O_2_·^−^ is slow in reaction with macromolecules, dismutation reaction of O_2_·^−^ produces hydrogen peroxide (H_2_O_2_), which can be transformed into ·OH and ^1^O_2_ [154]. Consequently, the three types of ROS (^1^O_2_, ·OH and O_2_·^−^) may coexist and contribute to the major oxidative stress in biological systems [29,105].

The electronic structure of metal-based NPs plays a key role in their ROS generation [155]. Metal-oxide NPs and metallic NPs have a distinct electronic structure, leading to their different ROS generation mechanisms. The electronic structure of metal-oxide NPs is characterized by the band gap (*E*_g_), which is essentially the energy interval between the valence band (*E*_v_) and the conduction band (*E*_c_), each of which has a high density of states [155]. Metallic NPs (e.g., Ag, Au and Ni) distinguish themselves from metal-oxide NPs by their unique surface plasmon resonance (SPR), which can significantly affect the photogenerated ROS by metallic NPs [156,157].

For metal-oxide NPs, the general principle is that when illuminated by light where the incident photon energy is greater than the band gap, the electrons (e^−^) of metal-oxide NPs are promoted across the band gap from the valence band to the conduction band, with the concomitant generation of a hole (*h*^+^) in the valence band [158]. Electrons in the conduction band and holes in the valence band exhibit high reducing and oxidizing power, respectively [159,160]. The photoexcited electrons can reduce molecular oxygen to O_2_·^−^ through a reductive process [158]. The hole can oxidize water and hydroxyl ions to generate ·OH through an oxidative process [161]. ^1^O_2_ is mostly produced indirectly from aqueous reactions of O_2_·^−^ [159].

**Figure 2:**
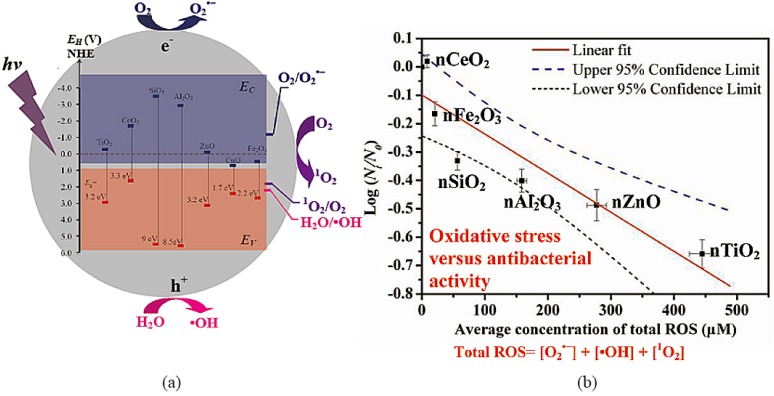
Mechanism of photogenerated ROS (**a**); and correlation with the antibacterial properties of metal-based NPs (**b**) (reproduced with permission from [29], Copyright American Chemical Society, 2012).

The production mechanism of a specific type or a combination of ROS in metal-oxide NP suspensions was previously elucidated by comparing the electronic structures (i.e., band edge energy levels) of the metal oxides with the redox potentials of various ROS generation [29,146,155,162]. For example, the *E*_c_ values of TiO_2_ and CeO_2_ are −0.28 and −1.69 eV with respect to normal hydrogen electrodes (NHE; all *E*_c_ and *E*_v_ values are with respect to NHE) [163,164], which are less than the *E*_H_ of O_2_/O_2_·^−^ (−0.2 eV) (Figure 2a) [29]. This indicates that the reductive power of the electrons in TiO_2_ and CeO_2_ is great enough to reduce O_2_ and lead to O_2_·^−^ generation. The reductive power of the electrons in CuO conduction band (*E*_c_ value of 0.69 eV) is insufficient to reduce O_2_ (Figure 2a) [163], so the CuO suspension could not generate O_2_·^−^. The *E*_v_ values of TiO_2_, ZnO and Fe_2_O_3_ are 2.92, 3.08 and 2.66 eV, respectively [163], which are greater than the *E*_H_ of H_2_O/·OH (2.2 eV) (Figure 2a) [29], Thus, the holes of these NPs can oxidize H_2_O and generate ·OH. The *E*_v_ value of CeO_2_ is 1.6 eV [164]; thus, CeO_2_ cannot generate ·OH (Figure 2a). Similarly, the *E*_v_ values of TiO_2_, ZnO, SiO_2_ and Al_2_O_3_ are 2.92, 3.08, 5.48 and 5.58 eV, respectively [163,165], which are greater than the *E*_H_ for ^1^O_2_/O_2_ (1.88 eV) (Figure 2a) [166]. Thus, these metal-oxide NPs have enough oxidizing power to facilitate ^1^O_2_ generation, which performed distinct antibacterial activities (Figure 2b).

However, comparison between the electronic structures of metal oxides with the redox potentials of various ROS generation cannot explain the ROS generation mechanisms for all metal-oxide NPs. For example, although the *E*_v_ values of CuO and Fe_2_O_3_ (2.39 and 2.66 eV) are greater than the *E*_H_ for ^1^O_2_/O_2_ (1.88 eV) [163], ^1^O_2_ is not detected in their suspensions. This is primarily because the released Cu^2+^ or Fe^2+^ could consume the produced ^1^O_2_. ZnO and Fe_2_O_3_ unexpectedly generated O_2_·^−^, which is probably because both of them are *n*-type semiconductors, whose conduction band could be bent upward when dispersed in water owing to the accumulation of positive charge within the space charge region of the Helmholtz layer [163]. Thus, their actual *E*_c_ could become lower than the *E*_H_ of O_2_/O_2_·^−^, which allows the generation of O_2_·^−^ by these NPs in water. In conclusion, the dissolution and *E*_c_ change of metal-oxide NPs in water should be taken into consideration when elucidating their ROS generation mechanisms.

Metallic NPs contain many free mobile electrons that can interact strongly with light by either absorbing or scattering the photons [156,157]. When metallic NPs are excited by light with wavelengths longer than the size of NPs, the oscillating electric field of the incoming radiation induces coherent collective oscillation of the free electrons (conduction band electrons) on the metal surface [156,157]. When the surface electron oscillation frequency is equal to the photon frequency, SPR is generated [156,157]. SPR induces a strong absorption of the incident photon energy, which can be transferred to O_2_ and lead to ^1^O_2_ generation [157,167,168]. The photoelectrons transferred to O_2_ are responsible for the generation of O_2_·^−^ [157], which can further promote the generation of ·OH under light irradiation [157,167,168]. Our group has demonstrated that Ag NPs produced both O_2_·^−^ and ·OH, but no detectable ^1^O_2_, whereas Ni NPs only produce ^1^O_2_ [31]. Not all three types of ROS were detected, which was primarily because the pronounced released Ag^+^ and Ni^2+^ led to the consumption of ROS [31]. Ni NPs are a type of magnetic transition metal and have damped plasmon resonance owing to their relatively large optical absorption coefficients [31,169]. This leads to less ROS generation by Ni NPs when compared to that for Ag NPs. Similar to metal-oxide NPs, the dissolution of metallic NPs could also influence ROS generation.

ROS generation of metal-based NPs could be influenced by many factors, including the characteristic parameters of NPs (e.g., particle size and surface coating), solution chemistry (e.g., pH and aqueous media) and experimental conditions (e.g., light conditions and NP concentration). In the following sections, the effects of the abovementioned parameters on ROS generation and types are discussed in detail.

#### 2.3.1. Effect of NP Concentrations on the ROS Generation

Many works have demonstrated that increasing NP concentrations led to their higher ROS generation concentrations [157,170,171]. A prevailing explanation for this is primarily because higher concentrations of NPs provided more surface area for reaction with oxygen and photons [157,170,171]. For example, higher concentrations of Au NPs showed a higher generation amount of ROS under UV or X-ray irradiation [157]. A significant increase in ROS generation was detected in Ag NP aqueous suspension when NP concentrations were increased from 10 to 50 mg·L^−1^ [170]. However, when the TiO_2_ concentration was increased from 0.1 to 1.0 g·L^−1^, the concentrations of ·OH only increased by approximately two times, but further increasing TiO_2_ concentration from 1.0 to 2.0 g·L^−1^ did not enhance the ·OH concentrations [161]. While the higher dosage of TiO_2_ provides more surface sites, it also decreases the effective light transmission into the NP suspension due to increased light scattering [161].

#### 2.3.2. Effect of Particle Size and Crystal Structure on the ROS Generation

As the particle size decreases, the surface areas of NPs exponentially increase, and a greater proportion of the atoms or molecules will be displayed on the surface and exposed to oxygen or photons [170,172,173]. In general, as the particle size decreases, ROS generation increases due to the increased surface areas and reactive sites for ROS generation [170,172,173]. For example, Misawa et al. investigated ROS production by Au (5–250 nm) under UV and X-ray irradiation, concluding that ROS generation concentrations almost linearly increased with the inverse of particle diameter (1/*d*) [157]. ROS generation of Ag NPs was also dependent on the size and increased with the decrease in their sizes [172].

Our group has compared ROS generation by a number of metal-oxide NPs and their bulk counterparts under UV-365 irradiation [29]. Metal-oxide NPs were found to yield more ROS than their bulk counterparts at equal-mass doses primarily due to larger surface areas of NPs providing more available reaction sites for UV absorption and oxygen exposure. Other potentially size-dependent properties (e.g., light absorption or scattering, defect sites and structural disorder) may also lead to a difference in photoactivity of NPs. The crystal phase of metal-based NPs also plays a critical role in their ROS generation [173]. TiO_2_ in anatase crystal structure produced higher amounts of intracellular ROS in *Escherichia coli* cells than possible in their rutile phase, thereby causing a propensity toward higher cytotoxicity [173].

#### 2.3.3. Effect of Surface Coating on the ROS Generation

Surface coating can change the characteristics of light absorption on metal-based NPs and will eventually enhance or reduce their ROS generation. It has been demonstrated that bare-Ag NPs generated O_2_·^−^ and ·OH; CIT-Ag NPs yielded only O_2_·^−^; whereas PVP-Ag NPs did not generate any type of ROS [156]. This was because PVP coatings shielded the active electron donor and acceptor sites on the NP surface, while the citrate coating, with its shorter chain length, less structural complexity and lower molecular weight, can inhibit the interaction between the electron donor and the Ag NP surface less efficiently than PVP coating [156]. In the biomedicine field, the photosensitizer was coated on the metal-based NP surface to enhance ROS generation for photodynamic treatment of cancer [167,168]. However, Wang et al. investigated that bio-extract capped Ag NPs decreased intracellular ROS production in hepatocellular liver carcinoma cells (HepG2) and human cervical cancer cells (HeLa), especially for ginger-Ag NPs and mint-Ag NPs. This could mostly be attributed to the antioxidant activity of biocapping agents on the surface of Ag NPs [29].

#### 2.3.4. Effect of Aqueous Medium Types on the ROS Generation

The physicochemical properties of the aqueous medium (i.e., pH and ionic strength) can significantly affect the ability of metal-based NPs to take advantage of ROS photogeneration [158,159]. It has been demonstrated that O_2_·^−^ production is enhanced at neutral and basic pH values that are more favorable to ROS formation through charge effects at the surface of TiO_2_ [162]. No significant pH effect on ·OH generation was observed in TiO_2_ aqueous suspension, as pH increased from 5.6 to 8.1 [161]. In addition, the solutes in aqueous media may affect the lifetime and reactivity of ROS [158], leading to the varied ROS generation types and concentrations of metal-based NPs in different aqueous media. Li et al. found that ZnO NPs generated three types of ROS (^1^O_2_, ·OH and O_2_·^−^) in deionized (DI) water, NaCl, phosphate-buffered saline (PBS) and minimal Davis (MD) medium, but only generated ^1^O_2_ and O_2_·^−^ in Luria–Bertani (LB) medium [159]. ROS production capacity could be reduced not only by the decreased surface area of ZnO NPs due to fast aggregation, but also by the organic components (e.g., citrate, glucose, tryptone and yeast extract) in the media [159].

Brunet et al. also demonstrated that TiO_2_ generated ^1^O_2_ in DI water, but not in the MD medium [158]. This resulted from the lesser reaction between ·OH and O_2_·^−^ (·OH + O_2_·^−^ → ^1^O_2_ + OH^−^) in MD as ·OH is consumed by citrate and glucose and more conversion in acidic conditions (2H^+^ + 2O_2_^·−^ → ^1^O_2_ + H_2_O_2_) [158]. Yet, the O_2_^·−^ concentration produced by TiO_2_ was significantly higher in the MD medium than in DI water because O_2_·^−^ production is more favorable at neutral and alkaline conditions through charge effects at the TiO_2_ surface [158]. In addition, the electron donors, such as hydroxyl and carboxylate groups on glucose and citrate, reduce the recombination of photoexcited electron-hole pairs [158].

#### 2.3.5. Effect of NOM on the ROS Generation

Sorption of NOM also interferes with ROS generation by metal-based NPs. However, controversial and inconsistent results on the effect of NOM on ROS generation by metal-based NPs exist, which merits more specific investigation. For example, Dasari and Hwang have demonstrated that both terrestrial HA and SRHA promote intracellular ROS generation by TiO_2_ in aquatic bacterial assemblages under natural sunlight irradiation. However, ROS generation by TiO_2_, ZnO and CuO NPs was inhibited by HA under natural sunlight irradiation, which was primarily because HA may act as effective quenchers of the produced ROS [174]. Another study has demonstrated that the intracellular oxidative stress of Ag NPs was not affected by the presence of HA since NOM could complex with Ag^+^ released by Ag NPs via a skeleton of HA comprised of alkyl and aromatic units [172]. The released metal ions could efficiently deactivate the triplet states of HA, leading to their decreased sensitization capacity for ROS generation by metal-based NPs [175]. Moreover, NOM can absorb photons in the 300–500 nm range of the solar spectrum because they contain conjugated unsaturated bonds and free electron pairs on heteroatoms [176,177,178]. Thus, they may act as a reducing buffer and UV filter for NPs [172].

#### 2.3.6. Effect of Light Condition and Temperature on the ROS Generation

Light exposure is an important environmental factor affecting ROS generation by metal-based NPs in water [156,159,179]. Previous studies have demonstrated that light exposure, such as irradiation by UV lamp, xenon lamp, solar and X-ray, could all promote ROS generation by metal-based NPs [156,159,179]. No measurable amount of ROS was detected by all metal-oxide and metallic NPs in the dark, while at least one type of ROS (^1^O_2_, ·OH and O_2_·^−^) was detected in their aqueous suspensions when exposed to UV-365 light [29,31,156]. This is because the light irradiation induces the generation of surface plasmon resonance (SPR) on the metallic particle surface and electron-hole pairs in metal-oxide NPs. Similarly, Masaki and Junko demonstrated that the ·OH and O_2_·^−^ generation by Au NPs under X-ray irradiation was enhanced by factors of 1.46 and 7.68 compared to that without light irradiation [157].

Light sources with different wavelengths vary ROS generation types and concentrations by metal-based NPs [156,157]. For example, under X-ray irradiation, Au NPs dispersed in water could produce ·OH and O_2_·^−^, while under UV irradiation, Au NPs could only generate O_2_·^−^ [157]. Another study demonstrated that bare-Ag NPs and CIT-Ag NPs did not generate any type of ROS under UV-254 and xenon lamp irradiation owing to the lesser extent of photoabsorption of the two types of Ag NPs; however, the bare-Ag NPs generated ·OH and O_2_·^−^, and the CIT-Ag NPs yielded O_2_·^−^ under UV-365 irradiation [156].

Solution temperature may also vary the ROS generation [161]. Increasing temperature generally enhances the mass transfer rates of dissolved oxygen to the reaction sites on the surfaces of metal-based NPs and also lowers the reaction activation energy, which leads to faster reaction kinetics according to the transition state theory. Higher temperature could result in more ·OH generation by TiO_2_ [161]. 

#### 2.3.7. Toxicity Implications of ROS Generation 

Bacterium is one of the most commonly-used model organisms for studying the toxicity implications of ROS from NPs. The photocatalytic ·OH concentration generated from TiO_2_ under UV illumination was linearly correlated with the rates of *E. coli* inactivation (*R*^2^ = 0.97), which indicates that ·OH is the primary oxidant species responsible for *E. coli* inactivation in the UV/TiO_2_ system [161]. However, not all ROS (i.e., ^1^O_2_ and O_2_·^−^) were taken into account when correlating the antibacterial activity of TiO_2_ in this study. It has been demonstrated that the average concentration of total ROS (^1^O_2_, ·OH and O_2_·^−^) generated by seven types of metal-oxide NPs under UV-365 irradiation followed the order of TiO_2_ > ZnO > Al_2_O_3_ > SiO_2_ > Fe_2_O_3_ > CeO_2_ > CuO [29]. A linear correlation was established between the average concentration of total ROS (^1^O_2_, ·OH, and O_2_·^−^) generated by these NPs and the bacterial survival rate of *E. coli* cells (*R*^2^ = 0.84). Li et al. found that the bacterial mortality rate monotonically increases with the increasing concentration of total ROS generated by ZnO NPs [159]. Another linear relationship was established with statistical significance between the total concentrations of the three types of ROS and the bacterial mortality rates of ZnO toward the *E. coli* cells in the five media (*R*^2^ = 0.92) [159]. Similarly, Ag NPs induced intracellular ROS generation in nitrifying bacteria, which correlated well with the antibacterial activity of Ag NPs (*R*^2^ = 0.86) [171]. However, there was a poor correlation between the inhibition of nitrifying bacteria by Ag NPs and their photocatalytic ROS concentrations (*R*^2^ = 0.53–0.72) [171]. 

The quantitative relationship between ROS generation by metal-based NPs and their toxicity has also been established in human cells. In addition to *E. coli* cells, the inverse correlation between declined cell viabilities and elevated ROS level was also observed in human HeLa cell, demonstrating that oxidative stress seems to be the key event by which CdS induces intracellular toxicity [180]. Shen et al. have demonstrated a strong inverse correlation between ZnO-induced cytotoxicity and O_2_·^−^ concentration (*R*^2^ = 0.80, *p* < 0.0001) in human immune cells, indicating a requirement for NP oxidative stress to precede cytotoxicity [181]. Dasari et al. [144] and Horev-Azaria et al. [182] investigated the toxicological effects of cobalt-ferrite (CoFe_2_O_4_) NPs on the viability of seven cell lines, which represented the different organs of the human body [182]. A high linear correlation (*R*^2^ = 0.97) was found between the toxicity of CoFe_2_O_4_ and the extent of ROS generation following their exposure to CoFe_2_O_4_ NPs, suggesting that oxidative stress is one possible mechanism for the toxicity of CoFe_2_O_4_ NPs [182].

## 3. Environmental Impacts of Metal-Based NPs on Aquatic Organisms

### 3.1. Adsorption of Metal-Based NPs at Cellular Interfaces

Adsorption is the first and an important step of interaction between metal-based NPs and aquatic species [37,183,184,185]. Since adsorption of NPs onto cellular interfaces is highly related to the toxicity of NPs, it is important to understand the mechanisms, equilibrium and kinetics of adsorption processes in the aquatic environment.

Interaction force boundary layer (IFBL) theory is a useful approach for describing adsorption kinetics of NPs at the interfaces [186]. According to this theory, the region adjacent to the surface can be divided into two different layers, including the inner layer (namely IFBL) and the outer layer. The inner layer thickness (δ_F_) corresponds to that of the EDL, while the outer layer thickness (δ_D_) corresponds to the diffusion boundary layer. The IFBL approach assumes that δ_D_ is much thicker than δ_F_ and that adsorption of NPs because interception and gravitational sedimentation is negligible. In fact, the total interaction energy between the NPs and cell surfaces, which determines the spontaneity of adsorption, can also be described by the EDLVO. Three major interfacial forces are involved in the calculation of surface interaction energy, which includes vdW, attraction, EDL repulsion and Lewis acid-base interaction (AB) [187]. EDLVO theory combined with the IFBL theory was applied in the calculation of the total interaction energy between NPs and cells [185], which was used to determine the adsorption rate constant (*k*_a_).

Once adsorption begins, the processes are mediated by several different colloidal forces. When NPs approach organism cells, the total interaction energy between NPs and cells is a function of the interaction distance and properties of cells and NPs, such as particle size and surface coating. Schwegmann et al. studied that the sorption process of NPs on *E. coli* was completed as short as a few minutes. In contrast, the concentration of NPs on *S. cerevisiae* increased continuously for 20 h [188]. Adsorption of NPs on cells was also shown to be particle size dependent [37,183,185]. The adsorption of large NPs (76 and 98 nm) on *E. coli* cells reached pseudo-adsorption equilibrium faster (30–40 min) than small NPs (60–90 min). While expressed as the number of adsorbed NPs per unit area of cells, it was found that small NPs had faster adsorption rates than large NPs [185], because smaller NPs tend to have a lower energy barrier [37], which made them easier to adsorb onto the cells; moreover, smaller NPs carry the greater surface energy, which induces more free energy (e.g., heat), which can be released through adsorption, and the thermodynamics is more favorable for small NP adsorption [185]. 

The surface properties of NPs and cells, surface coating, pH and ionic strength that could affect the zeta potential or surface charge largely determine the extent of the process [53,66,189]. The solution pH and ionic strength affect adsorption of NPs onto cells due to the change of surface charge [190,191,192]. Khan et al. found that the amount of adsorbed Ag NPs on bacterial cell surfaces decreased with an increase of pH with the maximum adsorption at pH 5. Moreover, a high concentration of NaCl could cover the surface of both NPs and bacterial cells and form an ionic shield that could decrease the attractive forces between NPs and bacterial cells [193]. Adsorption of Ag NPs onto bacterial cells was decreased with increasing NaCl concentration and then vanished until the concentration of NaCl was higher than 1 M, which was caused by the different zeta potentials of Gram-positive and Gram-negative bacterial cells [189]. Therefore, different adsorption behaviors of NPs can occur in different bacterial species even in an identical environment. 

### 3.2. Impacts of Metal-Based NPs on Single Aquatic Organisms

Following adsorption, NPs may accumulate on cell surfaces or undergo translocation into the intracellular environment via diffusion or endocytosis [188]. For instance, the radius of NPs is approximately 25–30 nm, as the rate of endocytosis reaches the maximum value [194]. The behavior of NPs at cellular interfaces will potentially induce adverse effects, which are discussed in details on plankton, fish and benthic organisms. 

#### 3.2.1. Aquatic Plants

The algal growth test is widely used to assess the risk of metal-based NPs in the aquatic environment. Generally, the metal ions released from metal-based NPs play an important role in the toxicity of NPs to aquatic organisms, especially for ZnO NPs. Franklin et al. reported that a significant toxicity of ZnO NPs to the freshwater algae *Pseudokirchneriella subcapitata* was solely caused by dissolved Zn [101]. However, both aggregation and ROS generation of ZnO and TiO_2_ NPs may induce toxicological impacts on *Chlorella* sp. [195]. Wang et al. pointed out that physical effects, including the TiO_2_ NP adsorption on the cell surface and algae entrapment inducing cell wall damage, caused a severe acute toxicity for algae *Phaeodactylum tricornutum* [196]. Furthermore, internalization is a common pathway for the uptake of NPs by algae [197]. TiO_2_ NPs inhibited the population growth of marine microalgae *Dunaliella tertiolecta* due to the NP internalization in algae cells inducing the destabilization of the DNA structure [198]. Leclerc and Wilkinson indicated that Ag bioaccumulation in *C. reinhardtii* increased significantly upon exposure to Ag NPs [199]. At the same exposed Ag^+^ concentration, the toxicity of Ag NPs to *C. reinhardtii* was higher than that of Ag^+^ as indicated by the photosynthetic yield of algae. Ag NPs induced more copper transport protein 2 (CTR2) upregulation than that exposed to the released Ag^+^ [199,200].

As for macrophytes, the Cu content in fronds of duckweeds exposed to CuO NPs was much higher than that exposed to a comparable dose of soluble Cu [106,201]. After taken up by the plant tissues, NPs may be translocated into vacuole (an organelle used to bind metals inside the cell) [202,203]. Furthermore, the growth of duckweeds was inhibited 50% by CuO NPs at the concentration of 1.0 mg·L^−1^. Exposure to CuO NPs induced a significant decrease of chlorophyll in plants [106], which may be caused by the reduced number of active photosystem II reaction centers and electron transport [201]. Both TiO_2_ NPs and ZnO NPs negatively affected the algal growth and chlorophyll a concentration at an early time [204].

#### 3.2.2. Zooplankton

Zooplanktons mostly feed on phytoplankton and, in turn, form food for animals at higher trophic levels, playing an important function in the food chain. NPs (such as TiO_2_ NPs and Al_2_O_3_ NPs) at high concentration (about 100 mg·L^−1^) were observed adhering to the external surface of *Daphnia magna* (*D. magna*) within 24 h from the beginning of the exposure [205]. Large amounts of NPs (i.e., TiO_2_ and Al_2_O_3_) were present in the gut tract of *D. magna* after being treated with NPs [205], because *D. magna* as a filter-feeder can ingest particles with sizes ranging from 0.9 to 18,000 µm^3^ [206]. Both TiO_2_ NPs and ZnO NPs negatively affected the algal growth and chlorophyll a concentration at an early time [204]. Meanwhile, bioconcentration factors (BCFs, L·kg^−1^) in *D. magna* were enhanced with increasing TiO_2_ NP concentrations with a low depuration [207]. Similarly, the Ag NP uptake was concentration dependent, but the efflux rate constants of Ag NPs in daphnia were much lower than those of Ag, also suggesting the difficulty of eliminating Ag NPs by daphnia [208]. In addition, *Ceriodaphnia dubia* from various treatments (1–50 mg·L^−1^ Fe_2_O_3_ NPs) accumulated Fe_2_O_3_ NPs with maximum values ranging from 0.043 to 0.133 μg·dubia^−1^ after being exposed for 6 h [209]. 

**Figure 3:**
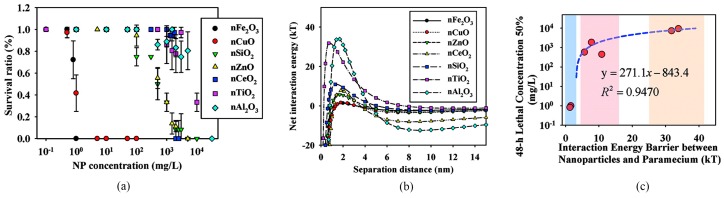
Surface interactions affect the toxicity of metal oxide NPs toward *Paramecium*: (**a**) survival ratios of *P. multimicronucleatum* after 48 h of exposure to NPs; (**b**) net interaction energy profiles between NPs and *P. multimicronucleatum*; (**c**) relationship of the magnitude of energy barrier and the 48-h *LC*_50_ of metal oxide NPs to *P. multimicronucleatum* (reproduced with permission from [213], Copyright American Chemical Society, 2012)

The composition of metal-based NPs significantly mediates their adverse ecotoxicological effects on freshwater zooplankton. Previous work showed that among three varieties of metal oxide NPs, ZnO NP suspension had the highest toxicity, while Al_2_O_3_ NPs were the least toxic to *D. magna*; meanwhile, both TiO_2_ and Al_2_O_3_ NPs were more toxic to *D. magna* than their bulk or large-sized particles [205]. Nevertheless, TiO_2_ NPs displayed no toxicity to *Daphnia pulex* adults, and Ag NPs and Cu NPs still caused toxicity with 48-h *LC*_50_s as low as 40 and 60 µg·L^−1^, respectively [210]. Another acute test showed the adverse effect of TiO_2_ NPs on the swimming behavior of aquatic animals [211]. Moreover, the acute toxicity of metal oxide NPs to *Paramecium multimicronucleatum* has shown that the 48-h *LC*_50_ values of these NPs decreased as follows: Al_2_O_3_ < TiO_2_ < CeO_2_ < ZnO < SiO_2_ < CuO < Fe_2_O_3_ NPs (Figure 3a), suggesting a positive correlation between the bonding strength of metal oxide NPs and the cell surface and the toxicity in unicellular organisms (Figure 3b,c) [212]. Furthermore, a recent study on the toxicity of CeO_2_ NPs to 14 ciliated protist species showed that the CeO_2_ NP adsorption on the protist surface rather than phylogenetical conservation induced the toxicity due to a negative correlation between *LC*_50_ values and the surface-to-volume ratio of protists [213]. In addition, based on the chronic exposure to 1 and 5 mg·L^−1^ of TiO_2_ NPs for 21 days, severe growth retardation, mortality and reproductive capacity reduction were all observed in *D. magna*. Even at a low concentration (0.1 mg·L^−1^), TiO_2_ NPs significantly reduced the number of offspring. When the TiO_2_ NPs were increased to 5 mg·L^−1^, the reproduction of *D. magna* was completely inhibited [207]. These results highlight the long-term risk of metal-based NPs to aquatic ecosystems.

#### 3.2.3. Nektonic Organisms (Fish)

Nektonic organisms, such as fish, are the active swimmers in aquatic systems (usually oceans or lakes). Fish may contribute to the transfer of contaminants, including NPs, to human beings and are one of the recommended groups for baseline toxicity studies of pollutants in the environment. It has been reported that zebrafish (*Danio rerio*) exposed to 0.1 and 1.0 mg·L^−1^ of TiO_2_ NPs could bioaccumulate TiO_2_ NPs with 25.38 and 181.38 of bioaccumulation factors (BCFs), respectively [214]. Similarly, after 25 days of exposure to 3 and 10 mg·L^−1^ of TiO_2_ NPs, the BCFs in carp (*Cyprinus carpio*) were found to be 675.5 and 595.4, respectively [215]. TiO_2_ NPs can have sublethal effects in fish (*Piaractus mesopotamicus*) [216], inhibit the growth of zebrafish (*D. rerio*), decrease the liver weight ratio in fish and accelerate the hatching of the larvae of zebrafish embryos [217,218]. Ferry et al. demonstrated that the corresponding concentration factor (*C*_f_) expressed as the ratio of the Au concentration (mg·kg^−1^) in the organisms to that in the water column (mg·kg^−1^) for *Cyprinodon variegatus* organs was 4.74 × 10^2^ [219]. The modified Au NPs killed all of the Japanese medaka (*Oryzias latipes*) within 24 h, showing significant toxicity to fish [220]. Jung et al. further found that the accumulation of CIT-Ag NPs and PVP-Ag NPs in Japanese medaka (*Oryzias latipes*) were lower than that of AgNO_3_ with respective BCF values [221]. The liver is the primary organ for bioaccumulation in Japanese medaka, which was independent of surface coating or released sliver ions [221]. In addition, Wang et al. found that salinity along with a nonionic surfactant (Tween 20) could promote the bioaccumulation of CIT-Ag NPs [222], indicating the importance of dispersion in bioavailability of Ag NPs in ionic environments.

#### 3.2.4. Benthos

In higher salinity (15‰–35‰) waters, such as marine environments, NPs likely aggregate and settle down in the sediment or benthic zones [223]. Benthic organisms are organisms that reside in the sediments and bottom waters and have the potential to interact with NPs or their aggregated forms [224,225,226,227]. However, so far, only a few studies have focused on the toxicity of metal-based NPs to benthic organisms. Buffet et al. reported that the Cu uptake was higher in clams (*S. plana*) exposed to soluble Cu than those exposed to CuO NPs, whereas in the worms (*H. diversicolor*), the opposite trend of Cu uptake was observed, owing to the different lifestyles of the species [224]. Moreover, Montes et al. demonstrated that mussels preferentially accumulated more Zn than Ce from the water column, but rejected more Ce than Zn in pseudofeces. The differences in NP solubility affects the NP uptake, excretion and accumulation in mussels [225]. Both ZnO and CuO NPs, except NiO NPs, were toxic to an estuarine amphipod (*Leptocheirus plumulosus*) [228]. It has been found that ZnO NPs had a different fate within the organs of benthic organisms. Furthermore, TiO_2_ NPs were mainly localized in endosomes and lysosomes, as well as the digestive system and significantly reduced the lysosomal membrane stability in the mussels [229]. Additionally, the aggregation of NPs may reduce bioavailability, but not eliminate it. For instance, the uptake rates in *Peringia ulvae* were lower than those reported for the freshwater snail *Lymnaea stagnalis*, probably because the bioavailability of Ag was reduced by the complexation of Ag^+^ in estuarine water and the aggregation of Ag NPs in saline conditions [230].

### 3.3. Impacts of Metal-Based NPs on Aquatic Organisms at Multiple Trophic Levels

The previous studies have shown that metal-based NPs can be ingested and accumulated in single aquatic organisms at different trophic levels from phytoplankton to benthos. Notably, it is highly possible that NPs are transferred from lower trophic organisms to higher trophic organisms through the food chain and biomagnified in the food web, considering that some aquatic organisms, such as fish and clams, are human food sources and also provide food for wildlife. 

Since 2008, many studies have been investigating the transfer of metal-based NPs in the food chain. For instance, Bouldin et al. verified the transfer of QDs from algae (*Pseudokirchneriella subcapitata*) to zooplankton (*Ceriodaphnia dubia*) after algae were treated with QDs for 24 h [231]. Werlin et al. reported that CdSe QDs accumulating in bacteria (*Pseudomonas aeruginosa*) were transferred to *Tetrahymena thermophile* [232], leading to a significant biomagnification. The high Au content in primary producer species consequently led to the high Au content in the primary consumer *D. magna* [233]. In addition, three-level trophic transfer of QDs in an aquatic food chain was demonstrated [234]. Significant amounts of TiO_2_ NPs were also detected in the dietary exposure groups, indicating that dietary intake may constitute a major route of trophic level [214]. Yet, negative biomagnification of TiO_2_ NPs was revealed in the simplified food chain due to the uptake and depuration profile for TiO_2_ NPs in the food [214]. However, a recent study still demonstrated the biomagnification of TiO_2_ NPs through microalga-scallop transfer [235]. QDs were transferred from ciliates to predatory rotifers through dietary uptake. Nonetheless, there was no evidence showing significant bioconcentration or biomagnification of QDs in this bacteria-ciliate-rotifer food web [236]. Similar transfer of Au NPs was found from the water column to the estuarine food web [219]. In a complex estuarine mesocosm, the photosynthetic biofilm fixed approximately 60% of the mass of NPs; Au was only found in the organ and gut of *Cyprinodon variegatus* without translocation to the circulatory system or absorbed by skin or gill contact; the filter feeders (*M. mercenaria*) taking up about 5% of the total NPs were the most effective sink for NPs [237], which is a potential route for metal-based NPs to enter the human food chain. Thus, it is clear that metallic NPs could be accumulated in aquatic organisms and transferred to different trophic levels, including alga, fish and benthic animals. However, there are still some controversial results for the biomagnification of NPs in aquatic environment, which deserves further investigation.

### 3.4. Genetic Impacts

Genetic effects may be produced by direct bindings of NPs with genetic materials (e.g., DNA and RNA), by indirect damage from ROS generated on NPs or by toxic ions released from soluble NPs [238,239]. The overall uptake of NPs that could reach the nucleus through diffusion across the nuclear membrane or be transported through nuclear pore complexes represents a danger of subsequent direct interaction with DNA molecules [240,241]. Particularly, single NPs of a small size could reach the nucleus through nuclear pores (~10 nm in diameter) [240,242]. Large NPs may also have access to DNA molecules when the nuclear membrane dissolves due to dividing cells during mitosis [243,244]. 

The significance of direct binding of NPs to DNA has not received as much attention as oxidative stress induced by NPs [43,96,241,245,246]. Our previous study showed that small QDs with a radius of 10 nm could permeate into bacterial cells and bind to DNA [247]. NP binding changed the normal conformation, as well as the local electrical properties of DNA molecules [247,248]. The binding of Au NPs also caused structural changes, including local denaturation and compaction of DNA [249]. Such changes may adversely interfere with the genetic functions of DNA, such as transcription, replication and repair processes that are crucial to maintaining the normal metabolism of a living cell [250,251,252,253,254]. Specifically, NPs that bind to DNA with a high affinity could inhibit the normal functions of some critical DNA-binding proteins, such as RNA polymerase and DNA polymerase, by occupying protein-binding sites and impeding the movement of protein along the DNA, which could result in competitive inhibition of genetic functions [250,251,253,255]. It has been reported that functionalized Au NPs completely inhibited DNA transcription in vitro owing to the electrostatic interaction of NPs with DNA [250,251,253]. A computational simulation study also showed that C_60_ strongly bound to DNA and might adversely impact the conformation and biological functions of DNA [254]. Furthermore, the binding of NPs to DNA might intervene in long-range charge transport through DNA and, thus, interfere with signaling processes [256]. Hence, the interaction between NPs and DNA has the potential to play important roles in the toxicity of NPs, and a complete elucidation or delineation of the underlying principles involved is essential to the safety of our ecosystem.

High-quality atomic force microscope (AFM) was employed to investigate the binding affinity of 12 types of NPs for DNA [248]. On the one hand, the QDs (+), Ag NPs, Fe_2_O_3_ NPs, Au NPs (citrate), CeO_2_ NPs, ZnO NPs and TiO_2_ NPs were observed to bind to DNA. On the other hand, the SiO_2_ NPs, Si NPs, QDs (−), Au NPs (COOH) and latex beads did not bind to DNA molecules. NPs with a high affinity for DNA may interfere with normal DNA functions. The binding of single NPs to DNA has the potential to change the DNA conformation dramatically, including DNA bending or looping capabilities in the presence of QDs (+) [257].

**Figure 4:**
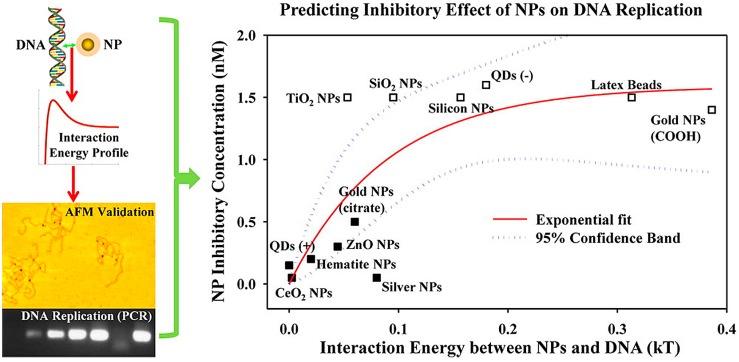
Relationship between the tested concentration of NPs significantly inhibiting DNA replication in vitro and the determined energy barrier between NPs and DNA (reprinted with permission from [258], Copyright American Chemical Society, 2013).

The PCR method can be employed to probe the effect of NPs on DNA replication [257]. The agarose gel electrophoresis revealed that QDs (+) completely inhibited DNA replication at the concentration of 0.15 nM, which agreed with a previous study wherein cationic QDs caused genotoxic effects [258]. Au NPs (citrate) affected DNA replication at 0.3 nM and completely impeded the replication process at the concentration of 0.5 nM. CeO_2_ NPs significantly inhibited DNA replication at 0.05 nM. ZnO suppressed the DNA replication process at 0.2 nM. This agrees with a previous study showing that Au NPs associated with DNA and subsequently induced DNA bending and strand separation [249]. In contrast, other NPs (e.g., QDs (−), TiO_2_ and SiO_2_ NPs) did not show any signs of inhibition at the highest concentration employed in this study (1.4–1.6 nM). The relation between the ability of NPs to inhibit DNA replication and the interaction energy between NPs and DNA can be computed by the DLVO theory [257]. NPs with predicted high binding affinity (i.e., low interaction energy barrier) with DNA molecules also had a high potential to inhibit DNA replication (Figure 4). This implied that: (1) the binding of NPs to DNA is likely an important mechanism for causing the adverse genetic effects of NPs; and (2) the interaction energy modeling approach could serve as a simple and effective tool for predicting the genetic effects of NPs induced by the direct binding activity of NPs with DNA.

## 4. Future Perspectives

NPs or ENMs exhibit remarkable properties that radically promote their applications [259]. Meanwhile, they pose emerging environmental impacts at an ever-increasing pace. Numerous crosscutting issues related to the safety assessment of ENMs have been identified that need to be addressed in order to promote sustainable growth of the nanotechnology industry. However, we are still facing many challenges to scientifically assess the environmental implications of metal-based NPs.

Firstly, most studies on the environmental behaviors of metal-based NPs have been conducted under laboratory conditions [29,81,130,156], which may hardly represent the realistic natural environments. Recently, some studies investigated the aggregation, dissolution and transformation of metal-based NPs in the natural water body by collecting lake water and seawater [133,260,261]. However, the knowledge on the environmental fate of metal-based NPs in the real environment is still limited. In fact, it is possible for NPs in the natural environment to be impacted simultaneously by various environmental factors, which makes the NP transformation more complicated. Thus, more experiments under realistic environmental conditions should be conducted to analyze the impact factors.

Secondly, a thorough understanding of the casual relationship between nanomaterial properties and toxicity is still largely unclear. Although many studies have been done on the implication of metal-based NPs to aquatic organisms, there is insufficient characterization of material properties and their relationship with the observed cytotoxicity and specific nanomaterial properties, such as the ROS generation. Thus, establishing a quantitative correlation between the ROS concentration and toxicity of metal-based NPs would facilitate the future evaluation and prediction on the toxicity of metal-based NPs.

Finally, many previous studies tend to attribute the toxicity of NPs to one or two major components of material properties (e.g., particle size effect) or solution factors. Nevertheless, it is worth noting that material properties are often interrelated and independent. For instance, when the particle size of NPs changes, other material properties (e.g., surface atom density, crystal facets, surface charge, surface hydrophobicity, mobility or diffusivity) may vary significantly. Moreover, it is well known that aquatic NPs are unstable, and after undergoing the above-mentioned processes, they may also undergo a transformation that will dramatically affect their size distribution and surface properties. However, tracking the dynamic aggregation or disaggregation to pinpoint the actual fractions of nanometer-sized ENMs (rather than aggregated or agglomerated particles) at cellular interfaces remains the most difficult task so far. At the same time, a tsunami of new ENMs should undergo testing or screening for toxicity.

## Figures and Tables

**Table 1:** Selected applications and intentional release of metal-based NPs.

Metal-Based NPs		Selected Applications	Release	References
Metallic NPs	Ag	Antimicrobial agent, wound healing, novel cancer therapy	Abrasion during use/washing, dissolution, disposal and recycling	[16,17,18]
	Au	Cellular imaging, photodynamic therapy, targeted drug delivery, biological sensors	Disposal and recycling	[19,20]
	Cu	Antimicrobial agent, catalyst, nanocomposite coating	Abrasion, disposal and recycling	[21,22]
	Fe	Environmental remediation	Intentional release	[21]
	Al	Drug delivery, wear-resistant coating additives	Abrasion during use/washing, disposal	[21]
	QDs	Medical imaging, targeted therapeutics, solar cells, telecommunications	During use, disposal	[23]
Metal oxide NPs	TiO_2_	Photocatalyst, antibacterial coating, paint, cosmetics, sunscreens	Abrasion, runoff, disposal and recycling	[22,24]
	CeO_2_	Fuel additive to decrease emissions, polishing and computer, chip manufacturing	Storm runoff, disposal and recycling	[24,25]
	ZnO	Sunscreen, skin protectant	Disposal	[22,23]
	CuO	Gas sensors, high-temperature superconductors, solar energy conversion, antimicrobial agent	During use, disposal	[26]
	Fe_2_O_3_	Biological imaging markers, environmental remediation	Disposal, intentional release	[21]
	SiO_2_	Electric and thermal insulators, adsorbents, filler materials, drug carriers, gene delivery	Abrasion during use, disposal	[21]

## References

[1] Alivisatos A.P. (1998). Semiconductor nanocrystals: New materials through control of size. Abstr. Pap. Am. Chem. Soc..

[2] Bailey R.E., Nie S.M. (2003). Alloyed semiconductor quantum dots: Tuning the optical properties without changing the particle size. J. Am. Chem. Soc..

[3] Zheng J., Zhang C.W., Dickson R.M. (2004). Highly fluorescent, water-soluble, size-tunable gold quantum dots. Phys. Rev. Lett..

[4] Barve A.V., Lee S.J., Noh S.K., Krishna S. (2010). Review of current progress in quantum dot infrared photodetectors. Laser Photonics Rev..

[5] Chan W.C.W., Nie S.M. (1998). Quantum dot bioconjugates for ultrasensitive nonisotopic detection. Science.

[6] Chang S., Zhou M., Grover C.P. (2004). Information coding and retrieving using fluorescent semiconductor nanocrystals for object identification. Opt. Express.

[7] Epstein H.A. (2011). Nanotechnology in cosmetic products. Skinmed.

[8] Kumar R., Anandan S., Hembram K., Rao T.N. (2014). Efficient ZnO-Based Visible-Light-Driven Photocatalyst for Antibacterial Applications. ACS Appl. Mater. Interfaces.

[9] Leung Y.H., Chan C.M.N., Ng A.M.C., Chan H.T., Chiang M.W.L., Djurisic A.B., Ng Y.H., Jim W.Y., Guo M.Y., Leung F.C.C. (2012). Antibacterial activity of ZnO nanoparticles with a modified surface under ambient illumination. Nanotechnology.

[10] Babu V.J., Kumar M.K., Nair A.S., Kheng T.L., Allakhverdiev S.I., Ramakrishna S. (2012). Visible light photocatalytic water splitting for hydrogen production from N-TiO_2_ rice grain shaped electrospun nanostructures. Int. J. Hydrogen. Energy.

[11] Kozlova E.A., Korobkina T.P., Vorontsov A.V. (2009). Overall water splitting over Pt/TiO_2_ Catalyst with Ce^3+^/Ce^4+^ shuttle charge transfer system. Int. J. Hydrogen. Energy.

[12] Chen C., Ma W., Zhao J. (2010). Semiconductor-mediated photodegradation of pollutants under visible-light irradiation. Chem. Soc. Rev..

[13] Kubacka A., Fernandez-Garcia M., Colon G. (2011). Advanced nanoarchitectures for solar photocatalytic applications. Chem. Rev..

[14] Wiesner M.R., Lowry G.V., Alvarez P., Dionysiou D., Biswas P. (2006). Assessing the Risks of Manufactured Nanomaterials. Environ. Sci. Technol..

[15] Lowry G.V., Hotze E.M., Bernhardt E.S., Dionysiou D.D., Pedersen J.A., Wiesner M.R., Xing B. (2010). Environmental occurrences, behavior, fate, and ecological effects of nanomaterials: An introduction to the special series. J. Environ. Qual..

[16] Gottschalk F., Sonderer T., Scholz R.W., Nowack B. (2009). Modeled environmental concentrations of engineered nanomaterials (TiO_2_, ZnO, Ag, CNT, Fullerenes) for different regions. Environ. Sci. Technol..

[17] Fabrega J., Luoma S.N., Tyler C.R., Galloway T.S., Lead J.R. (2011). Silver nanoparticles: Behaviour and effects in the aquatic environment. Environ. Int..

[18] Benn T.M., Westerhoff P. (2008). Nanoparticle silver released into water from commercially available sock fabrics. Environ. Sci. Technol..

[19] Mueller N.C., Nowack B. (2008). Exposure modeling of engineered nanoparticles in the environment. Environ. Sci. Technol..

[20] Huang X., Jain P.K., El-Sayed I.H., El-Sayed M.A. (2007). Gold nanoparticles: Interesting optical properties and recent applications in cancer diagnostics and therapy. Nanomedicine.

[21] Schrand A.M., Rahman M.F., Hussain S.M., Schlager J.J., Smith D.A., Ali S.F. (2010). Metal-based nanoparticles and their toxicity assessment. Wires Nanomed. Nanobiotechnol..

[22] Liu R., Lun N., Qi Y.X., Zhu H.L., Bai Y.J., Bi J.Q. (2011). Synthesis of hollow carbon sphere/ZnO@C composite as a light-weight microwave absorber. J. Phys. D Appl. Phys..

[23] Klaine S.J., Alvarez P.J., Batley G.E., Fernandes T.F., Handy R.D., Lyon D.Y., Mahendra S., McLaughlin M.J., Lead J.R. (2008). Nanomaterials in the environment: Behavior, fate, bioavailability, and effects. Environ. Toxicol. Chem..

[24] Gottschalk F., Nowack B. (2011). The release of engineered nanomaterials to the environment. J. Environ. Monit..

[25] Gottschalk F., Sun T., Nowack B. (2013). Environmental concentrations of engineered nanomaterials: Review of modeling and analytical studies. Environ. Pollut..

[26] Ren G., Hu D., Cheng E.W., Vargas-Reus M.A., Reip P., Allaker R.P. (2009). Characterisation of copper oxide nanoparticles for antimicrobial applications. Int. J. Antimicrob. Agents.

[27] Coll C., Notter D., Gottschalk F., Sun T., Som C., Nowack B. (2016). Probabilistic environmental risk assessment of five nanomaterials (nano-TiO_2_, nano-Ag, nano-ZnO, CNT, and fullerenes). Nanotoxicology.

[28] Li Y., Zhang W., Li K., Yao Y., Niu J., Chen Y. (2012). Oxidative dissolution of polymer-coated CdSe/ZnS quantum dots under UV irradiation: Mechanisms and kinetics. Environ. Pollut..

[29] Li Y., Zhang W., Niu J., Chen Y. (2012). Mechanism of photogenerated reactive oxygen species and correlation with the antibacterial properties of engineered metal-oxide nanoparticles. ACS Nano.

[30] Li Y., Zhang W., Niu J., Chen Y. (2013). Surface-coating-dependent dissolution, aggregation, and reactive oxygen species (ROS) generation of silver nanoparticles under different irradiation conditions. Environ. Sci. Technol..

[31] Zhang W., Li Y., Niu J., Chen Y. (2013). Photogeneration of reactive oxygen species on uncoated silver, gold, nickel, and silicon nanoparticles and their antibacterial effects. Langmuir.

[32] Van Hoecke K., Quik J.T., Mankiewicz-Boczek J., De Schamphelaere K.A., Elsaesser A., Van der Meeren P., Barnes C., McKerr G., Howard C.V., Van de Meent D. (2009). Fate and effects of CeO_2_ nanoparticles in aquatic ecotoxicity tests. Environ. Sci. Technol..

[33] Wang Z.Y., Li J., Zhao J., Xing B.S. (2011). Toxicity and internalization of CuO nanoparticles to prokaryotic alga *Microcystis aeruginosa* as affected by dissolved organic matter. Environ. Sci. Technol..

[34] Faust J.J., Zhang W., Koeneman B.A., Chen Y.S., Capco D.G. (2012). Commenting on the effects of surface treated- and non-surface treated TiO_2_ in the Caco-2 cell model. Part. Fibre Toxicol..

[35] Kalive M., Zhang W., Chen Y.S., Capco D.G. (2012). Human intestinal epithelial cells exhibit a cellular response indicating a potential toxicity upon exposure to hematite nanoparticles. Cell Biol. Toxicol..

[36] Watari F., Takashi N., Yokoyama A., Uo M., Akasaka T., Sato Y., Abe S., Totsuka Y., Tohji K. (2009). Material nanosizing effect on living organisms: Non-specific, biointeractive, physical size effects. J. R. Soc. Interface.

[37] Zhang W., Kalive M., Capco D.G., Chen Y.S. (2010). Adsorption of hematite nanoparticles onto Caco-2 cells and the cellular impairments: Effect of particle size. Nanotechnology.

[38] Geisler-Lee J., Wang Q., Yao Y., Zhang W., Geisler M., Li K.G., Huang Y., Chen Y.S., Kolmakov A., Ma X.M. (2013). Phytotoxicity, accumulation and transport of silver nanoparticles by *Arabidopsis thaliana*. Nanotoxicology.

[39] Zhang W., Crittenden J., Li K.G., Chen Y.S. (2012). Attachment efficiency of nanoparticle aggregation in aqueous dispersions: Modeling and experimental validation. Environ. Sci. Technol..

[40] Colvin V.L. (2003). The potential environmental impact of engineered nanomaterials. Nat. Biotechnol..

[41] Donaldson K., Tran L., Jimenez L.A., Duffin R., Newby D.E., Mills N., MacNee W., Stone V. (2005). Combustion-derived nanoparticles: A review of their toxicology following inhalation exposure. Part. Fibre Toxicol..

[42] Pujalte I., Passagne I., Brouillaud B., Treguer M., Durand E., Ohayon-Courtes C., L'Azou B. (2011). Cytotoxicity and oxidative stress induced by different metallic nanoparticles on human kidney cells. Part. Fibre Toxicol..

[43] Nel A., Xia T., Madler L., Li N. (2006). Toxic potential of materials at the nanolevel. Science.

[44] Zhang W., Yao Y., Li K., Huang Y., Chen Y. (2011). Influence of dissolved oxygen on aggregation kinetics of citrate-coated silver nanoparticles. Environ. Pollut..

[45] Chen K.L., Elimelech M. (2006). Aggregation and deposition kinetics of fullerene (C60) nanoparticles. Langmuir.

[46] Li K., Zhang W., Huang Y., Chen Y. (2011). Aggregation kinetics of CeO_2_ nanoparticles in KCl and CaCl_2_ solutions: Measurements and modeling. J. Nanopart. Res..

[47] Lowry G.V., Gregory K.B., Apte S.C., Lead J.R. (2012). Transformations of nanomaterials in the environment. Environ. Sci. Technol..

[48] Waychunas G.A., Kim C.S., Banfield J.F. (2005). Nanoparticulate iron oxide minerals in soils and sediments: Unique properties and contaminant scavenging mechanisms. J. Nanopart. Res..

[49] Elimelech M., Jia X., Gregory J., Williams R. (1995). Surface interaction potentials. Particle Deposition & Aggregation: Measurement, Modelling and Simulation.

[50] Bhattacharjee S., Elimelech M. (1997). Surface element integration: A novel technique for evaluation of DLVO interaction between a particle and a flat plate. J. Collioid Interface Sci..

[51] Hoek E., Agarwal G.K. (2006). Extended DLVO interactions between spherical particles and rough surfaces. J. Collioid Interface Sci..

[52] Fernández-García M., Rodriguez J.A. (2006). Metal Oxide Nanoparticles. Encyclopedia of Inorganic Chemistry.

[53] Hotze E.M., Phenrat T., Lowry G.V. (2010). Nanoparticle aggregation: Challenges to understanding transport and reactivity in the environment. J. Environ. Qual..

[54] Buettner K.M., Rinciog C.I., Mylon S.E. (2010). Aggregation kinetics of cerium oxide nanoparticles in monovalent and divalent electrolytes. Colloids Surf. A.

[55] Zhou D.X., Ji Z.X., Jiang X.M., Dunphy D.R., Brinker J., Keller A.A. (2013). Influence of material properties on TiO_2_ nanoparticle agglomeration. PLoS ONE.

[56] Perron H., Domain C., Roques J., Drot R., Simoni E., Catalette H. (2007). Optimisation of accurate rutile TiO_2_ (110), (100), (101) and (001) surface models from periodic DFT calculations. Theor. Chem. Acc..

[57] Huynh K.A., Chen K.L. (2011). Aggregation kinetics of citrate and polyvinylpyrrolidone coated silver nanoparticles in monovalent and divalent electrolyte solutions. Environ. Sci. Technol..

[58] Allen H.J., Impellitteri C.A., Macke D.A., Heckman J.L., Poynton H.C., Lazorchak J.M., Govindaswamy S., Roose D.L., Nadagouda M.N. (2010). Effects from filtration, capping agents, and presence/absence of food on the toxicity of silver nanoparticles to daphnia magna. Environ. Toxicol. Chem..

[59] Kittler S., Greulich C., Koeller M., Epple M. (2009). Synthesis of PVP-coated silver nanoparticles and their biological activity towards human mesenchymal stem cells. Materialwiss. Werkst..

[60] Levard C., Greulich C., Koeller M., Epple M. (2011). Sulfidation processes of PVP-coated silver nanoparticles in aqueous solution: Impact on dissolution rate. Environ. Sci. Technol..

[61] Li X., Lenhart J.J. (2012). Aggregation and dissolution of silver nanoparticles in natural surface water. Environ. Sci. Technol..

[62] Ma R., Levard C., Marinakos S.M., Cheng Y., Liu J., Michel F.M., Brown G.E., Lowry G.V. (2012). Size-controlled dissolution of organic-coated silver nanoparticles. Environ. Sci. Technol..

[63] Hezinger A.F.E., Teßmar J., Göpferich A. (2008). Polymer coating of quantum dots—A powerful tool toward diagnostics and sensorics. Eur. J. Pharm. Biopharm..

[64] Hydutsky B.W., Mack E.J., Beckerman B.B., Skluzacek J.M., Mallouk T.E. (2007). Optimization of nano- and microiron transport through sand columns using polyelectrolyte mixtures. Environ. Sci. Technol..

[65] Mayya K.S., Schoeler B., Caruso F. (2003). Preparation and organization of nanoscale polyelectrolyte-coated gold nanoparticles. Adv. Funct. Mater..

[66] Phenrat T., Saleh N., Sirk K., Kim H.-J., Tilton R., Lowry G. (2008). Stabilization of aqueous nanoscale zerovalent iron dispersions by anionic polyelectrolytes: Adsorbed anionic polyelectrolyte layer properties and their effect on aggregation and sedimentation. J. Nanopart. Res..

[67] Rosen M.J., Kunjappu J.T. (2012). Adsorption of Surface-Active Agents at Interfaces: The Electrical Double Layer. Surfactants and Interfacial Phenomena.

[68] Kvitek L., Panacek A., Soukupova J., Kolar M., Vecerova R., Prucek R., Holecova M., Zboril R. (2008). Effect of surfactants and polymers on stability and antibacterial activity of silver nanoparticles (NPs). J. Phys. Chem. C.

[69] Ellis L.-J.A., Valsami-Jones E., Lead J.R., Baalousha M. (2016). Impact of surface coating and environmental conditions on the fate and transport of silver nanoparticles in the aquatic environment. Sci. Total Environ..

[70] Raza G., Amjad M., Kaur I., Wen D. (2016). Stability and aggregation kinetics of titania nanomaterials under environmentally realistic conditions. Environ. Sci. Technol..

[71] Yang D., Rochette J., Sacher E. (2005). Spectroscopic evidence for π-π interaction between poly(diallyl dimethylammonium) chloride and multiwalled carbon nanotubes. J. Phys. Chem. B.

[72] Chen H., Wang Y., Dong S., Wang E. (2006). One-step preparation and characterization of PDDA-protected gold nanoparticles. Polymer.

[73] Lodeiro P., Achterberg E.P., Pampín J., Affatati A., El-Shahawi M.S. (2016). Silver nanoparticles coated with natural polysaccharides as models to study AgNP aggregation kinetics using UV-Visible spectrophotometry upon discharge in complex environments. Sci. Total Environ..

[74] Vaisman L., Wagner H.D., Marom G. (2006). The role of surfactants in dispersion of carbon nanotubes. Adv. Colloid Interface Sci..

[75] Li X., Lenhart J.J., Walker H.W. (2011). Aggregation kinetics and dissolution of coated silver nanoparticles. Langmuir.

[76] Dederichs T., Möller M., Weichold O. (2009). colloidal stability of hydrophobic nanoparticles in ionic surfactant solutions: Definition of the critical dispersion concentration. Langmuir.

[77] Hermansson M. (1999). The DLVO theory in microbial adhesion. Colloid Surf. B.

[78] Bian S.W., Mudunkotuwa I.A., Rupasinghe T., Grassian V.H. (2011). Aggregation and dissolution of 4 nm ZnO nanoparticles in aqueous environments: Influence of pH, ionic strength, size, and adsorption of humic acid. Langmuir.

[79] Van Hoecke K., De Schamphelaere K.A., Van der Meeren P., Smagghe G., Janssen C.R. (2011). Aggregation and ecotoxicity of CeO_2_ nanoparticles in synthetic and natural waters with variable pH, organic matter concentration and ionic strength. Environ. Pollut..

[80] Guzman K.A., Finnegan M.P., Banfield J.F. (2006). Influence of surface potential on aggregation and transport of titania nanoparticles. Environ. Sci. Technol..

[81] French R.A., Jacobson A.R., Kim B., Isley S.L., Penn R.L., Baveye P.C. (2009). Influence of ionic strength, pH, and cation valence on aggregation kinetics of titanium dioxide nanoparticles. Environ. Sci. Technol..

[82] Lin D., Drew Story S., Walker S.L., Huang Q., Cai P. (2016). Influence of extracellular polymeric substances on the aggregation kinetics of TiO_2_ nanoparticles. Water Res..

[83] Sheng A., Liu F., Xie N., Liu J. (2016). Impact of proteins on aggregation kinetics and adsorption ability of hematite nanoparticles in aqueous dispersions. Environ. Sci. Technol..

[84] Louie S.M., Spielman-Sun E.R., Small M.J., Tilton R.D., Lowry G.V. (2015). Correlation of the physicochemical properties of natural organic matter samples from different sources to their effects on gold nanoparticle aggregation in monovalent electrolyte. Environ. Sci. Technol..

[85] Louie S.M., Tilton R.D., Lowry G.V. (2013). Effects of molecular weight distribution and chemical properties of natural organic matter on gold nanoparticle aggregation. Environ. Sci. Technol..

[86] Zhou D., Keller A.A. (2010). Role of morphology in the aggregation kinetics of ZnO nanoparticles. Water Res..

[87] Pallem V.L., Stretz H.A., Wells M.J. (2009). Evaluating aggregation of gold nanoparticles and humic substances using fluorescence spectroscopy. Environ. Sci. Technol..

[88] Wang Z., Quik J.T., Song L., van den Brandhof E.J., Wouterse M., Peijnenburg W.J. (2015). Humic substances alleviate the aquatic toxicity of PVP-coated silver nanoparticles to organisms of different trophic levels. Environ. Toxicol. Chem..

[89] Zhu M., Wang H., Keller A.A., Wang T., Li F. (2014). The effect of humic acid on the aggregation of titanium dioxide nanoparticles under different pH and ionic strengths. Sci. Total Environ..

[90] Deonarine A., Lau B.L., Aiken G.R., Ryan J.N., Hsu-Kim H. (2011). Effects of humic substances on precipitation and aggregation of zinc sulfide nanoparticles. Environ. Sci. Technol..

[91] Nason J.A., McDowell S.A., Callahan T.W. (2012). Effects of natural organic matter type and concentration on the aggregation of citrate-stabilized gold nanoparticles. J. Environ. Monit..

[92] Afshinnia K., Gibson I., Merrifield R., Baalousha M. (2016). The concentration-dependent aggregation of Ag NPs induced by cystine. Sci. Total Environ..

[93] Garcia-Garcia S., Wold S., Jonsson M. (2009). Effects of temperature on the stability of colloidal montmorillonite particles at different pH and ionic strength. Appl. Clay Sci..

[94] Garcia-Garcia S., Jonsson M., Wold S. (2006). Temperature effect on the stability of bentonite colloids in water. J. Collioid Interface Sci..

[95] Grasso D., Subramaniam K., Butkus M., Strevett K., Bergendahl J. (2002). A review of non-DLVO interactions in environmental colloidal systems. Rev. Environ. Sci. Biotechnol..

[96] Nel A.E., Madler L., Velegol D., Xia T., Hoek E.M.V., Somasundaran P., Klaessig F., Castranova V., Thompson M. (2009). Understanding biophysicochemical interactions at the nano-bio interface. Nat. Mater..

[97] Pierres A., Benoliel A.-M., Zhu C., Bongrand P. (2001). Diffusion of microspheres in shear flow near a wall: Use to measure binding rates between attached molecules. Biophys. J..

[98] Datsko T.Y., Zelentsov V.I. (2009). Dependence of the surface charge and the fluorine adsorption by γ-aluminum oxide on the solution temperature. Surf. Eng. Appl. Electrochem..

[99] Rodriguez K., Araujo M. (2006). Temperature and pressure effects on zeta potential values of reservoir minerals. J. Colloid Interface Sci..

[100] Kittler S., Greulich C., Diendorf J., Köller M., Epple M. (2010). Toxicity of silver nanoparticles increases during storage because of slow dissolution under release of silver ions. Chem. Mater..

[101] Franklin N.M., Rogers N.J., Apte S.C., Batley G.E., Gadd G.E., Casey P.S. (2007). Comparative toxicity of nanoparticulate ZnO, bulk ZnO, and ZnCl_2_ to a freshwater microalga (*Pseudokirchneriella subcapitata*): The importance of particle solubility. Environ. Sci. Technol..

[102] Yang X.Y., Gondikas A.P., Marinakos S.M., Auffan M., Liu J., Hsu-Kim H., Meyer J.N. (2012). Mechanism of silver nanoparticle toxicity is dependent on dissolved silver and surface coating in *Caenorhabditis elegans*. Environ. Sci. Technol..

[103] Zhang W., Yao Y., Sullivan N., Chen Y. (2011). Modeling the primary size effects of citrate-coated silver nanoparticles on their ion release kinetics. Environ. Sci. Technol..

[104] Brun N.R., Lenz M., Wehrli B., Fent K. (2014). Comparative effects of zinc oxide nanoparticles and dissolved zinc on zebrafish embryos and eleuthero-embryos: Importance of zinc ions. Sci. Total Environ..

[105] Xia T., Kovochich M., Liong M., Madler L., Gilbert B., Shi H., Yeh J.I., Zink J.I., Nel A.E. (2008). Comparison of the mechanism of toxicity of zinc oxide and cerium oxide nanoparticles based on dissolution and oxidative stress properties. ACS Nano.

[106] Shi J.Y., Abid A.D., Kennedy I.M., Hristova K.R., Silk W.K. (2011). To duckweeds (*Landoltia punctata*), nanoparticulate copper oxide is more inhibitory than the soluble copper in the bulk solution. Environ. Pollut..

[107] Vogelsberger W., Schmidt J., Roelofs F. (2008). Dissolution kinetics of oxidic nanoparticles: The observation of an unusual behaviour. Colloid Surf. A.

[108] Schmidt J., Vogelsberger W. (2006). Dissolution kinetics of titanium dioxide nanoparticles: The observation of an unusual kinetic size effect. J. Phys. Chem. B.

[109] Borm P., Klaessig F.C., Landry T.D., Moudgil B., Pauluhn J., Thomas K., Trottier R., Wood S. (2006). Research strategies for safety evaluation of nanomaterials, part V: Role of dissolution in biological fate and effects of nanoscale particles. Toxicol. Sci..

[110] Elzey S., Grassian V.H. (2010). Agglomeration, isolation and dissolution of commercially manufactured silver nanoparticles in aqueous environments. J. Nanopart. Res..

[111] Ho C.M., Yau S.K.W., Lok C.N., So M.H., Che C.M. (2010). Oxidative dissolution of silver nanoparticles by biologically relevant oxidants: A kinetic and mechanistic study. Chemistry.

[112] Misra S.K., Dybowska A., Berhanu D., Croteau M.N., Luoma S.N., Boccaccini A.R., Valsami-Jones E. (2012). Isotopically modified nanoparticles for enhanced detection in bioaccumulation studies. Environ. Sci. Technol..

[113] Odzak N., Kistler D., Behra R., Sigg L. (2014). Dissolution of metal and metal oxide nanoparticles in aqueous media. Environ. Pollut..

[114] Siy J.T., Bartl M.H. (2010). Insights into reversible dissolution of colloidal CdSe nanocrystal quantum dots. Chem. Mater..

[115] Diedrich T., Dybowska A., Schott J., Valsarni-Jones E., Oelkers E.H. (2012). The dissolution rates of SiO_2_ nanoparticles as a function of particle size. Environ. Sci. Technol..

[116] Gilbert B., Huang F., Zhang H., Waychunas G.A., Banfield J.F. (2004). Nanoparticles: Strained and stiff. Science.

[117] Luo W.H., Hu W.Y., Xiao S.F. (2008). Size effect on the thermodynamic properties of silver nanoparticles. J. Phys. Chem. C.

[118] Mortimer M., Kasemets K., Kahru A. (2010). Toxicity of ZnO and CuO nanoparticles to ciliated protozoa *Tetrahymena thermophila*. Toxicology.

[119] Misra S.K., Nuseibeh S., Dybowska A., Berhanu D., Tetley T.D., Valsami-Jones E. (2014). Comparative study using spheres, rods and spindle-shaped nanoplatelets on dispersion stability, dissolution and toxicity of CuO nanomaterials. Nanotoxicology.

[120] Chen Z.W., Waje M., Li W.Z., Yan Y.S. (2007). Supportless Pt and PtPd nanotubes as electrocatalysts for oxygen-reduction reactions. Angew. Chem. Int. Ed..

[121] Gunawan C., Teoh W.Y., Marquis C.P., Amal R. (2011). Cytotoxic origin of copper(II) oxide nanoparticles: Comparative studies with micron-sized particles, leachate, and metal salts. ACS Nano.

[122] Liu J., Sonshine D.A., Shervani S., Hurt R.H. (2010). Controlled release of biologically active silver from nanosilver surfaces. ACS Nano.

[123] Liu J., Aruguete D.A., Jinschek J.R., Rimstidt J.D., Hochella M.F. (2008). The non-oxidative dissolution of galena nanocrystals: Insights into mineral dissolution rates as a function of grain size, shape, and aggregation state. Geochim. Cosmochim. Acta.

[124] Tsukahara T., Hibara A., Ikeda Y., Kitamori T. (2007). NMR study of water molecules confined in extended nanospaces. Angew. Chem..

[125] Hoshi N., Nakamura M., Yoshida C., Yamada Y., Kameyama M., Mizumoto Y. (2016). In-situ high-speed AFM of shape-controlled Pt nanoparticles in electrochemical environments: Structural effects on the dissolution mechanism. Electrochem. Commun..

[126] Gelabert A., Sivry Y., Ferrari R., Akrout A., Cordier L., Nowak S., Menguy N., Benedetti M.F. (2014). Uncoated and coated ZnO nanoparticle life cycle in synthetic seawater. Environ. Toxicol. Chem..

[127] Baumann J., Koser J., Arndt D., Filser J. (2014). The coating makes the difference: Acute effects of iron oxide nanoparticles on Daphnia magna. Sci. Total Environ..

[128] Domingos R.F., Simon D.F., Hauser C., Wilkinson K.J. (2011). Bioaccumulation and effects of CdTe/CdS quantum dots on *Chlamydomonas reinhardtii*—Nanoparticles or the free ions?. Environ. Sci. Technol..

[129] Miao A.J., Zhang X.Y., Luo Z., Chen C.S., Chin W.C., Santschi P.H., Quigg A. (2010). Zinc oxide-engineered nanoparticles: Dissolution and toxicity to marine phytoplankton. Environ. Toxicol. Chem..

[130] Dahle J.T., Livi K., Arai Y. (2015). Effects of pH and phosphate on CeO nanoparticle dissolution. Chemosphere.

[131] Chen M., Wang L.Y., Han J.T., Zhang J.Y., Li Z.Y., Qian D.J. (2006). Preparation and study of polyacryamide-stabilized silver nanoparticles through a one-pot process. J. Phys. Chem. B.

[132] Li X., Lenhart J.J., Walker H.W. (2010). Dissolution-accompanied aggregation kinetics of silver nanoparticles. Langmuir.

[133] Zhang L., Li J., Yang K., Liu J., Lin D. (2016). Physicochemical transformation and algal toxicity of engineered nanoparticles in surface water samples. Environ. Pollut..

[134] Levard C., Hotze E.M., Lowry G.V., Brown G.E. (2012). Environmental transformations of silver nanoparticles: Impact on stability and toxicity. Environ. Sci. Technol..

[135] Taghavy A., Mittelman A., Wang Y., Pennell K.D., Abriola L.M. (2013). Mathematical modeling of the transport and dissolution of citrate-stabilized silver nanoparticles in porous media. Environ. Sci. Technol..

[136] Collin B., Tsyusko O.V., Starnes D.L., Unrine J.M. (2016). Effect of natural organic matter on dissolution and toxicity of sulfidized silver nanoparticles to *Caenorhabditis elegans*. Environ. Sci. Nano.

[137] Yang K., Lin D., Xing B. (2009). Interactions of humic acid with nanosized inorganic oxides. Langmuir.

[138] Akaighe N., Depner S.W., Banerjee S., Sharma V.K., Sohn M. (2012). The effects of monovalent and divalent cations on the stability of silver nanoparticles formed from direct reduction of silver ions by Suwannee River humic acid/natural organic matter. Sci. Total Environ..

[139] Mudunkotuwa I.A., Rupasinghe T., Wu C.M., Grassian V.H. (2012). Dissolution of ZnO nanoparticles at circumneutral pH: A study of size effects in the presence and absence of citric acid. Langmuir.

[140] Gondikas A.P., Morris A., Reinsch B.C., Marinakos S.M., Lowry G.V., Hsu-Kim H. (2012). Cysteine-induced modifications of zero-valent silver nanomaterials: Implications for particle surface chemistry, aggregation, dissolution, and silver speciation. Environ. Sci. Technol..

[141] Lv J., Zhang S., Luo L., Han W., Zhang J., Yang K., Christie P. (2012). Dissolution and microstructural transformation of ZnO nanoparticles under the influence of phosphate. Environ. Sci. Technol..

[142] Garg S., Rong H., Miller C.J., Waite T.D. (2016). Oxidative dissolution of silver nanoparticles by chlorine: Implications to silver nanoparticle fate and toxicity. Environ. Sci. Technol..

[143] Gorham J.M., MacCuspie R.I., Klein K.L., Fairbrother D.H., Holbrook R.D. (2012). UV-induced photochemical transformations of citrate-capped silver nanoparticle suspensions. J. Nanopart. Res..

[144] Kim Y.J., Yu M., Park H.O., Yang S.I. (2010). Comparative study of cytotoxicity, oxidative stress and genotoxicity induced by silica nanomaterials in human neuronal cell line. Mol. Cell. Toxicol..

[145] Xia T., Kovochich M., Brant J., Hotze M., Sempf J., Oberley T., Sioutas C., Yeh J.I., Wiesner M.R., Nel A.E. (2006). Comparison of the abilities of ambient and manufactured nanoparticles to induce cellular toxicity according to an oxidative stress paradigm. Nano Lett..

[146] Kaweeteerawat C., Ivask A., Liu R., Zhang H., Chang C.H., Low-Kam C., Fischer H., Ji Z., Pokhrel S., Cohen Y. (2015). Toxicity of metal oxide nanoparticles in *Escherichia coli* correlates with conduction band and hydration energies. Environ. Sci. Technol..

[147] Dasari T.P., Pathakoti K., Hwang H.M. (2013). Determination of the mechanism of photoinduced toxicity of selected metal oxide nanoparticles (ZnO, CuO, Co_3_O_4_ and TiO_2_) to *E. coli* bacteria. J. Environ. Sci. China.

[148] Karunakaran C., Rajeswari V., Gomathisankar P. (2010). Antibacterial and photocatalytic activities of sonochemically prepared ZnO and Ag-ZnO. J. Alloys Compd..

[149] Seven O., Dindar B., Aydemir S., Metin D., Ozinel M.A., Icli S. (2004). Solar photocatalytic disinfection of a group of bacteria and fungi aqueous suspensions with TiO_2_, ZnO and Sahara desert dust. J. Photochem. Photobiol. A.

[150] Du J., Gebicki J.M. (2004). Proteins are major initial cell targets of hydroxyl free radicals. Int. J. Biochem. Cell Biol..

[151] Fujii M., Usui M., Hayashi S., Gross E., Kovalev D., Kunzner N., Diener J., Timoshenko V.Y. (2004). Chemical reaction mediated by excited states of Si nanocrystals—Singlet oxygen formation in solution. J. Appl. Phys..

[152] Bakalova R., Ohba H., Zhelev Z., Ishikawa M., Baba Y. (2004). Quantum dots as photosensitizers?. Nat. Biotechnol..

[153] Watts R.J., Washington D., Howsawkeng J., Loge F.J., Teel A.L. (2003). Comparative toxicity of hydrogen peroxide, hydroxyl radicals, and superoxide anion to *Escherichia coli*. Adv. Environ. Res..

[154] Irwin F. (1986). Biological effects of the superoxide radical. Arch. Biochem. Biophys..

[155] Gratzel M. (2001). Photoelectrochemical cells. Nature.

[156] Batley G.E., Kirby J.K., McLaughlin M.J. (2013). Fate and risks of nanomaterials in aquatic and terrestrial environments. Acc. Chem. Res..

[157] Misawa M., Takahashi J. (2011). Generation of reactive oxygen species induced by gold nanoparticles under X-ray and UV Irradiations. Nanomed. Nanotechnol..

[158] Brunet L., Lyon D.Y., Hotze E.M., Alvarez P.J.J., Wiesner M.R. (2009). Comparative photoactivity and antibacterial properties of C_60_ fullerenes and titanium dioxide nanoparticles. Environ. Sci. Technol..

[159] Li Y., Niu J., Zhang W., Zhang L., Shang E. (2014). Influence of aqueous media on the ROS-mediated toxicity of ZnO nanoparticles toward green fluorescent protein-expressing *Escherichia coli* under UV-365 irradiation. Langmuir.

[160] Lin H.F., Liao S.C., Hung S.W. (2005). The dc thermal plasma synthesis of ZnO nanoparticles for visible-light photocatalyst. J. Photochem. Photobiol. A.

[161] Cho M., Chung H., Choi W., Yoon J. (2004). Linear correlation between inactivation of *E. coli* and OH radical concentration in TiO_2_ photocatalytic disinfection. Water Res..

[162] Kormann C., Bahnemann D.W., Hoffmann M.R. (1991). Photolysis of chloroform and other organic-molecules in aqueous TiO_2_ suspensions. Environ. Sci. Technol..

[163] Xu Y., Schoonen M.A.A. (2000). The absolute energy positions of conduction and valence bands of selected semiconducting minerals. Am. Mineral..

[164] Preisler E.J., Marsh O.J., Beach R.A., McGill T.C. (2001). Stability of cerium oxide on silicon studied by X-ray photoelectron spectroscopy. J. Vac. Sci. Technol. B.

[165] DiStefano T.H., Eastman D.E. (1971). The band edge of amorphous SiO_2_ by photoinjection and photoconductivity measurements. Solid State Commun..

[166] Maurette M.T., Oliveros E., Infelta P.P., Ramsteiner K., Braun A.M. (1983). Singlet oxygen and superoxide: Experimental differentiation and analysis. Helv. Chim. Acta.

[167] Khaing Oo M.K., Yang Y., Hu Y., Gomez M., Du H., Wang H. (2012). Gold nanoparticle-enhanced and size-dependent generation of reactive oxygen species from protoporphyrin IX. ACS Nano.

[168] Wieder M.E., Hone D.C., Cook M.J., Handsley M.M., Gavrilovic J., Russell D.A. (2006). Intracellular photodynamic therapy with photosensitizer-nanoparticle conjugates: Cancer therapy using a ‘Trojan horse’. Photochem. Photobiol. Sci..

[169] Wang L., Clavero C., Huba Z., Carroll K.J., Carpenter E.E., Gu D., Lukaszew R.A. (2011). Plasmonics and enhanced magneto-optics in core-shell co-Ag nanoparticles. Nano Lett..

[170] Carlson C., Hussain S.M., Schrand A.M., Braydich-Stolle L.K., Hess K.L., Jones R.L., Schlager J.J. (2008). Unique cellular interaction of silver nanoparticles: Size-dependent generation of reactive oxygen species. J. Phys. Chem. B.

[171] Choi O., Hu Z. (2008). Size dependent and reactive oxygen species related nanosilver toxicity to nitrifying bacteria. Environ. Sci. Technol..

[172] Bae E., Park H.-J., Yoon J., Kim Y., Choi K., Yi J. (2011). Bacterial uptake of silver nanoparticles in the presence of humic acid and AgNO_3_. Korean J. Chem. Eng..

[173] Lin X., Li J., Ma S., Liu G., Yang K., Tong M., Lin D. (2014). Toxicity of TiO_2_ nanoparticles to *Escherichia coli*: Effects of particle size, crystal phase and water chemistry. PLoS ONE.

[174] Lin D., Ji J., Long Z., Yang K., Wu F. (2012). The influence of dissolved and surface-bound humic acid on the toxicity of TiO_2_ nanoparticles to *Chlorella* sp.. Water Res..

[175] Carlos L., Cipollone M., Soria D.B., Moreno M.S., Ogilby P.R., Einschlag F.S.G., Martire D.O. (2012). The effect of humic acid binding to magnetite nanoparticles on the photogeneration of reactive oxygen species. Sep. Purif. Technol..

[176] Dong M.M., Rosario-Ortiz F.L. (2012). Photochemical formation of hydroxyl radical from effluent organic matter. Environ. Sci. Technol..

[177] De Laurentiis E., Buoso S., Maurino V., Minero C., Vione D. (2013). Optical and photochemical characterization of chromophoric dissolved organic matter from lakes in Terra Nova Bay, Antarctica. Evidence of considerable photoreactivity in an extreme environment. Environ. Sci. Technol..

[178] Lee E., Glover C.M., Rosario-Ortiz F.L. (2013). Photochemical formation of hydroxyl radical from effluent organic matter: Role of composition. Environ. Sci. Technol..

[179] Dasari T.P., Hwang H.M. (2013). Effect of humic acids and sunlight on the cytotoxicity of engineered zinc oxide and titanium dioxide nanoparticles to a river bacterial assemblage. J. Environ. Sci. China.

[180] Hossain S.T., Mukherjee S.K. (2013). Toxicity of cadmium sulfide (CdS) nanoparticles against *Escherichia coil* and HeLa cells. J. Hazard. Mater..

[181] Shen C., James S.A., de Jonge M.D., Turney T.W., Wright P.F.A., Feltis B.N. (2013). Relating Cytotoxicity, Zinc ions, and reactive oxygen in ZnO nanoparticle exposed human immune cells. Toxicol. Sci..

[182] Horev-Azaria L., Baldi G., Beno D., Bonacchi D., Golla-Schindler U., Kirkpatrick J.C., Kolle S., Landsiedel R., Maimon O., Marche P.N. (2013). Predictive toxicology of cobalt ferrite nanoparticles: Comparative in vitro study of different cellular models using methods of knowledge discovery from data. Part. Fibre Toxicol..

[183] Chithrani B.D., Ghazani A.A., Chan W.C.W. (2006). Determining the size and shape dependence of gold nanoparticle uptake into mammalian cells. Nano Lett..

[184] Patil S., Sandberg A., Heckert E., Self W., Seal S. (2007). Protein adsorption and cellular uptake of cerium oxide nanoparticles as a function of zeta potential. Biomaterials.

[185] Zhang W., Rittmann B., Chen Y. (2011). Size effects on adsorption of hematite nanoparticles on *E. coli* cells. Environ. Sci. Technol..

[186] Petosa A.R., Jaisi D.P., Quevedo I.R., Elimelech M., Tufenkji N. (2010). Aggregation and deposition of engineered nanomaterials in aquatic environments: Role of physicochemical interactions. Environ. Sci. Technol..

[187] Zhang W., Zhang X. (2015). Adsorption of MS2 on oxide nanoparticles affects chlorine disinfection and solar inactivation. Water Res..

[188] Schwegmann H., Feitz A.J., Frimmel F.H. (2010). Influence of the zeta potential on the sorption and toxicity of iron oxide nanoparticles on *S. cerevisiae* and *E. coli*. J. Colloid Interface Sci..

[189] Khan S.S., Mukherjee A., Chandrasekaran N. (2011). Studies on interaction of colloidal silver nanoparticles (SNPs) with five different bacterial species. Colloids Surf. B.

[190] Jiang W., Mashayekhi H., Xing B. (2009). Bacterial toxicity comparison between nano- and micro-scaled oxide particles. Environ. Pollut..

[191] Peng Z.G., Hidajat K., Uddin M.S. (2004). Adsorption of bovine serum albumin on nanosized magnetic particles. J. Colloid Interface Sci..

[192] Li B., Logan B.E. (2004). Bacterial adhesion to glass and metal-oxide surfaces. Colloids Surf. B.

[193] Liu H.-S., Wang Y.-C., Chen W.-Y. (1995). The sorption of lysozyme and ribonuclease onto ferromagnetic nickel powder 1. Adsorption of single components. Colloids Surf. B.

[194] Zhang S., Li J., Lykotrafitis G., Bao G., Suresh S. (2009). Size-dependent endocytosis of nanoparticles. Adv. Mater..

[195] Ji J., Long Z.F., Lin D.H. (2011). Toxicity of oxide nanoparticles to the green algae *Chlorella* sp.. Chem. Eng. J..

[196] Wang Y., Zhu X., Lao Y., Lv X., Tao Y., Huang B., Wang J., Zhou J., Cai Z. (2016). TiO_2_ nanoparticles in the marine environment: Physical effects responsible for the toxicity on algae *Phaeodactylum tricornutum*. Sci. Total Environ..

[197] Miao A.J., Luo Z., Chen C.S., Chin W.C., Santschi P.H., Quigg A. (2010). Intracellular uptake: A possible mechanism for silver engineered nanoparticle toxicity to a freshwater alga Ochromonas danica. PLoS ONE.

[198] Schiavo S., Oliviero M., Miglietta M., Rametta G., Manzo S. (2016). Genotoxic and cytotoxic effects of ZnO nanoparticles for *Dunaliella tertiolecta* and comparison with SiO_2_ and TiO_2_ effects at population growth inhibition levels. Sci. Total Environ..

[199] Leclerc S., Wilkinson K.J. (2014). Bioaccumulation of nanosilver by *Chlamydomonas reinhardtii*-nanoparticle or the free ion?. Environ. Sci. Technol..

[200] Navarro E., Piccapietra F., Wagner B., Marconi F., Kaegi R., Odzak N., Sigg L., Behra R. (2008). Toxicity of silver nanoparticles to *Chlamydomonas reinhardtii*. Environ. Sci. Technol..

[201] Perreault F., Samadani M., Dewez D. (2014). Effect of soluble copper released from copper oxide nanoparticles solubilisation on growth and photosynthetic processes of *Lemna gibba* L.. Nanotoxicology.

[202] Hall J.L. (2002). Cellular mechanisms for heavy metal detoxification and tolerance. J. Exp. Bot..

[203] Glenn J.B., Klaine S.J. (2013). Abiotic and biotic factors that influence the bioavailability of gold nanoparticles to aquatic macrophytes. Environ. Sci. Technol..

[204] Hazeem L.J., Bououdina M., Rashdan S., Brunet L., Slomianny C., Boukherroub R. (2016). Cumulative effect of zinc oxide and titanium oxide nanoparticles on growth and chlorophyll a content of *Picochlorum* sp.. Environ. Sci. Pollut. Res..

[205] Zhu X., Zhu L., Chen Y., Tian S. (2009). Acute toxicities of six manufactured nanomaterial suspensions to *Daphnia magna*. J. Nanopart. Res..

[206] Adams L.K., Lyon D.Y., McIntosh A., Alvarez P.J. (2006). Comparative toxicity of nano-scale TiO_2_, SiO_2_ and ZnO water suspensions. Water Sci. Technol..

[207] Zhu X., Chang Y., Chen Y. (2010). Toxicity and bioaccumulation of TiO_2_ nanoparticle aggregates in *Daphnia magna*. Chemosphere.

[208] Zhao C.M., Wang W.X. (2010). Biokinetic uptake and efflux of silver nanoparticles in *Daphnia magna*. Environ. Sci. Technol..

[209] Hu J., Wang D., Wang J. (2012). Bioaccumulation of Fe_2_O_3_(magnetic) nanoparticles in *Ceriodaphnia dubia*. Environ. Pollut..

[210] Griffitt R.J., Luo J., Gao J., Bonzongo J.C., Barber D.S. (2008). Effects of particle composition and species on toxicity of metallic nanomaterials in aquatic organisms. Environ. Toxicol. Chem..

[211] Noss C., Dabrunz A., Rosenfeldt R.R., Lorke A., Schulz R. (2013). Three-dimensional analysis of the swimming behavior of *Daphnia magna* exposed to nanosized titanium dioxide. PLoS ONE.

[212] Li K., Chen Y., Zhang W., Pu Z., Jiang L. (2012). Surface interactions affect the toxicity of engineered metal oxide nanoparticles toward *Paramecium*. Chem. Res. Toxicol..

[213] Zhang W., Pu Z., Du S., Chen Y., Jiang L. (2016). Fate of engineered cerium oxide nanoparticles in an aquatic environment and their toxicity toward 14 ciliated protist species. Environ. Pollut..

[214] Zhu X., Wang J., Zhang X., Chang Y., Chen Y. (2010). Trophic transfer of TiO_2_ nanoparticles from *Daphnia* to zebrafish in a simplified freshwater food chain. Chemosphere.

[215] Zhang X., Sun H., Zhang Z., Niu Q., Chen Y., Crittenden J.C. (2007). Enhanced bioaccumulation of cadmium in carp in the presence of titanium dioxide nanoparticles. Chemosphere.

[216] Clemente Z., Castro V.L., Feitosa L.O., Lima R., Jonsson C.M., Maia A.H., Fraceto L.F. (2013). Fish exposure to nano-TiO_2_ under different experimental conditions: Methodological aspects for nanoecotoxicology investigations. Sci. Total Environ..

[217] Chen J.Y., Dong X., Xin Y.Y., Zhao M.R. (2011). Effects of titanium dioxide nano-particles on growth and some histological parameters of zebrafish (*Danio rerio*) after a long-term exposure. Aquat. Toxicol..

[218] Clemente Z., Castro V.L.S.S., Moura M.A.M., Jonsson C.M., Fraceto L.F. (2014). Toxicity assessment of TiO_2_ nanoparticles in zebrafish embryos under different exposure conditions. Aquat. Toxicol..

[219] Ferry J.L., Craig P., Hexel C., Sisco P., Frey R., Pennington P.L., Fulton M.H., Scott I.G., Decho A.W., Kashiwada S. (2009). Transfer of gold nanoparticles from the water column to the estuarine food web. Nat. Nanotechnol..

[220] Zhu Z.J., Carboni R., Quercio M.J., Yan B., Miranda O.R., Anderton D.L., Arcaro K.F., Rotello V.M., Vachet R.W. (2010). Surface properties dictate uptake, distribution, excretion, and toxicity of nanoparticles in fish. Small.

[221] Jung Y.J., Kim K.T., Kim J.Y., Yang S.Y., Lee B.G., Kim S.D. (2014). Bioconcentration and distribution of silver nanoparticles in Japanese medaka (*Oryzias latipes*). J. Hazard. Mater..

[222] Wang J., Wang W.X. (2014). Salinity influences on the uptake of silver nanoparticles and silver nitrate by marine medaka (*Oryzias melastigma*). Environ. Toxicol. Chem..

[223] Zhu X., Cai Z. (2012). Behavior and effect of manufactured nanomaterials in the marine environment. Integr. Environ. Assess. Manag..

[224] Buffet P.E., Richard M., Caupos F., Vergnoux A., Perrein-Ettajani H., Luna-Acosta A., Akcha F., Amiard J.C., Amiard-Triquet C., Guibbolini M. (2013). A mesocosm study of fate and effects of CuO nanoparticles on endobenthic species (*Scrobicularia plana*, *Hediste diversicolor*). Environ. Sci. Technol..

[225] Montes M.O., Hanna S.K., Lenihan H.S., Keller A.A. (2012). Uptake, accumulation, and biotransformation of metal oxide nanoparticles by a marine suspension-feeder. J. Hazard. Mater..

[226] Galloway T., Lewis C., Dolciotti I., Johnston B.D., Moger J., Regoli F. (2010). Sublethal toxicity of nano-titanium dioxide and carbon nanotubes in a sediment dwelling marine polychaete. Environ. Pollut..

[227] Buffet P.E., Amiard-Triquet C., Dybowska A., Risso-de Faverney C., Guibbolini M., Valsami-Jones E., Mouneyrac C. (2012). Fate of isotopically labeled zinc oxide nanoparticles in sediment and effects on two endobenthic species, the clam Scrobicularia plana and the ragworm *Hediste diversicolor*. Ecotoxicol. Environ. Saf..

[228] Hanna S.K., Miller R.J., Zhou D., Keller A.A., Lenihan H.S. (2013). Accumulation and toxicity of metal oxide nanoparticles in a soft-sediment estuarine amphipod. Aquat. Toxicol..

[229] Jimeno-Romero A., Oron M., Cajaraville M.P., Soto M., Marigómez I. (2016). Nanoparticle size and combined toxicity of TiO_2_ and DSLS (surfactant) contribute to lysosomal responses in digestive cells of mussels exposed to TiO_2_ nanoparticles. Nanotoxicology.

[230] Khan F.R., Laycock A., Dybowska A., Larner F., Smith B.D., Rainbow P.S., Luoma S.N., Rehkamper M., Valsami-Jones E. (2013). Stable isotope tracer to determine uptake and efflux dynamics of ZnO nano- and bulk particles and dissolved Zn to an estuarine snail. Environ. Sci. Technol..

[231] Bouldin J.L., Ingle T.M., Sengupta A., Alexander R., Hannigan R.E., Buchanan R.A. (2008). Aqueous toxicity and food chain transfer of quantum dots in freshwater algae and *Ceriodaphnia dubia*. Environ. Toxicol. Chem..

[232] Werlin R., Priester J.H., Mielke R.E., Kramer S., Jackson S., Stoimenov P.K., Stucky G.D., Cherr G.N., Orias E., Holden P.A. (2011). Biomagnification of cadmium selenide quantum dots in a simple experimental microbial food chain. Nat. Nanotechnol..

[233] Lee W.M., Yoon S.J., Shin Y.J., An Y.J. (2015). Trophic transfer of gold nanoparticles from *Euglena gracilis* or *Chlamydomonas reinhardtii* to *Daphnia magna*. Environ. Pollut..

[234] Lee W.M., An Y.J. (2015). Evidence of three-level trophic transfer of quantum dots in an aquatic food chain by using bioimaging. Nanotoxicology.

[235] Wang Z., Xia B., Chen B., Sun X., Zhu L., Zhao J., Du P., Xing B. (2017). Trophic transfer of TiO_2_ nanoparticles from marine microalga (*Nitzschia closterium*) to scallop (*Chlamys farreri*) and related toxicity. Environ. Sci. Nano.

[236] Holbrook R.D., Murphy K.E., Morrow J.B., Cole K.D. (2008). Trophic transfer of nanoparticles in a simplified invertebrate food web. Nat. Nanotechnol..

[237] Decho A.W. (1990). Microbial exopolymer secretions in ocean environments—Their role(s) in food webs and marine processes. Oceanogr. Mar. Biol..

[238] Kisin E.R., Murray A.R., Keane M.J., Shi X.C., Schwegler-Berry D., Gorelik O., Arepalli S., Castranova V., Wallace W.E., Kagan V.E. (2007). Single-walled carbon nanotubes: Geno- and cytotoxic effects in lung fibroblast V79 cells. J. Toxicol. Environ. Health A.

[239] Magdolenova Z., Collins A., Kumar A., Dhawan A., Stone V., Dusinska M. (2014). Mechanisms of genotoxicity. A review of in vitro and in vivo studies with engineered nanoparticles. Nanotoxicology.

[240] Gu Y.J., Cheng J., Lin C.C., Lam Y.W., Cheng S.H., Wong W.T. (2009). Nuclear penetration of surface functionalized gold nanoparticles. Toxicol. Appl. Pharmacol..

[241] Soenen S.J., Rivera-Gil P., Montenegro J.-M., Parak W.J., De Smedt S.C., Braeckmans K. (2011). Cellular toxicity of inorganic nanoparticles: Common aspects and guidelines for improved nanotoxicity evaluation. Nano Today.

[242] Barillet S., Jugan M.L., Laye M., Leconte Y., Herlin-Boime N., Reynaud C., Carriere M. (2010). In vitro evaluation of SiC nanoparticles impact on A549 pulmonary cells: Cyto-, genotoxicity and oxidative stress. Toxicol. Lett..

[243] Liang X.J., Chen C., Zhao Y., Jia L., Wang P.C. (2008). Biopharmaceutics and therapeutic potential of engineered nanomaterials. Curr. Drug Metab..

[244] Singh N., Manshian B., Jenkins G.J., Griffiths S.M., Williams P.M., Maffeis T.G., Wright C.J., Doak S.H. (2009). NanoGenotoxicology: The DNA damaging potential of engineered nanomaterials. Biomaterials.

[245] Kumar A., Pandey A.K., Singh S.S., Shanker R., Dhawan A. (2011). Engineered ZnO and TiO_2_ nanoparticles induce oxidative stress and DNA damage leading to reduced viability of *Escherichia coli*. Free Radic. Biol. Med..

[246] Sanvicens N., Marco M.P. (2008). Multifunctional nanoparticles—Properties and prospects for their use in human medicine. Trends Biotechnol..

[247] Zhang W., Yao Y., Chen Y. (2011). Imaging and quantifying the morphology and nanoelectrical properties of quantum dot nanoparticles interacting with DNA. J. Phys. Chem. C.

[248] Li K., Zhang W., Chen Y. (2013). Quantum dot binding to DNA: Single-molecule imaging with atomic force microscopy. Biotechnol. J..

[249] Railsback J.G., Singh A., Pearce R.C., McKnight T.E., Collazo R., Sitar Z., Yingling Y.G., Melechko A.V. (2012). Weakly Charged Cationic Nanoparticles Induce DNA Bending and Strand Separation. Adv. Mater..

[250] Han G., Chari N.S., Verma A., Hong R., Martin C.T., Rotello V.M. (2005). Controlled recovery of the transcription of nanoparticle-bound DNA by intracellular concentrations of glutathione. Bioconjug. Chem..

[251] McIntosh C.M., Esposito E.A., Boal A.K., Simard J.M., Martin C.T., Rotello V.M. (2001). Inhibition of DNA Transcription Using Cationic Mixed Monolayer Protected Gold Clusters. J. Am. Chem. Soc..

[252] Takenaka S., Yamashita K., Takagi M., Hatta T., Tanaka A., Tsuge O. (1999). Study of the DNA interaction with water-soluble cationic fullerene derivatives. Chem. Lett..

[253] You C.-C., Chompoosor A., Rotello V.M. (2007). The biomacromolecule-nanoparticle interface. Nano Today.

[254] Zhao X., Striolo A., Cummings P.T. (2005). C60 binds to and deforms nucleotides. Biophys. J..

[255] Johnston H.J., Hutchison G., Christensen F.M., Peters S., Hankin S., Stone V. (2010). A review of the in vivo and in vitro toxicity of silver and gold particulates: Particle attributes and biological mechanisms responsible for the observed toxicity. Crit. Rev. Toxicol..

[256] Shukla S., Sastry M. (2009). Probing differential Ag^+^-nucleobase interactions with isothermal titration calorimetry (ITC): Towards patterned DNA metallization. Nanoscale.

[257] Li K., Zhao X., B K.H., Du S., Chen Y. (2013). Nanoparticles inhibit DNA replication by binding to DNA: Modeling and experimental validation. ACS Nano.

[258] Tiwari D.K., Jin T., Behari J. (2011). Bio-distribution and toxicity assessment of intravenously injected anti-HER2 antibody conjugated CdSe/ZnS quantum dots in Wistar rats. Int. J. Nanomed..

[259] Baun A., Hartmann N.B., Grieger K., Kusk K.O. (2008). Ecotoxicity of engineered nanoparticles to aquatic invertebrates: A brief review and recommendations for future toxicity testing. Ecotoxicology.

[260] Conway J.R., Adeleye A.S., Gardea-Torresdey J., Keller A.A. (2015). Aggregation, dissolution, and transformation of copper nanoparticles in natural waters. Environ. Sci. Technol..

[261] Lu D., Liu Q., Zhang T., Cai Y., Yin Y., Jiang G. (2016). Stable silver isotope fractionation in the natural transformation process of silver nanoparticles. Nat. Nano.

